# Predicting oil accumulation by fruit image processing and linear models in traditional and super high-density olive cultivars

**DOI:** 10.3389/fpls.2024.1456800

**Published:** 2024-10-25

**Authors:** Giuseppe Montanaro, Antonio Carlomagno, Angelo Petrozza, Francesco Cellini, Ioanna Manolikaki, Georgios Koubouris, Vitale Nuzzo

**Affiliations:** ^1^ Department of Agricultural, Forest, Food, and Environmental Sciences, Potenza, Italy; ^2^ Agenzia Lucana di Sviluppo e Innovazione in Agricoltura (ALSIA) Centro Ricerche Metapontum Agrobios, Metaponto, MT, Italy; ^3^ Hellenic Agricultural Organization ELGO-DIMITRA, Institute of Olive Tree, Subtropical Crops and Viticulture, Chania, Greece

**Keywords:** colorimetric indexes, hysteresis, *Olea europaea* L., plantation systems, SHD, NIR, RGB

## Abstract

The paper focuses on the seasonal oil accumulation in traditional and super-high density (SHD) olive plantations and its modelling employing image-based linear models. For these purposes, at 7-10-day intervals, fruit samples (cultivar Arbequina, Fasola, Frantoio, Koroneiki, Leccino, Maiatica) were pictured and images segmented to extract the Red (R), Green (G), and Blue (B) mean pixel values which were re-arranged in 35 RGB-derived colorimetric indexes (*CIs*). After imaging, the samples were crushed and oil concentration was determined (NIR). The analysis of the correlation between oil and *CIs* revealed a differential hysteretic behavior depending on the covariates (*CI* and cultivar). The hysteresis area (*Hyst*) was then quantified and used to rank the *CIs* under the hypothesis that *CIs* with the maximum or minimum *Hyst* had the highest correlation coefficient and were the most suitable predictors within a general linear model. The results show that the predictors selected according to Hyst-based criteria had high accuracy as determined using a Global Performance Indicator (GPI) accounting for various performance metrics (*R* ^2^, RSME, MAE). The use of a general linear model here presented is a new computational option integrating current methods mostly based on artificial neural networks. RGB-based image phenotyping can effectively predict key quality traits in olive fruit supporting the transition of the olive sector towards a digital agriculture domain.

## 1 Introduction

Olive crop is considered the cornerstone of Mediterranean agriculture for history, culture, nutrition, and economy. At present, about 90% of the world’s olive groves, covering over 10 million hectares, are located in the Mediterranean region and it is nowadays globally expanding also towards the Southern Hemisphere (Torres et al., 2017), likely because table olives and oil are increasingly recognized as functional food within the context of a healthy lifestyle (Accardi et al., 2016; Agrawal et al., 2017). Aside from that geographical expansion, olive crops are undergoing a business model transformation including plantation design and management (Lo Bianco et al., 2021). Following this, the most common traditional olive groves (70-200 trees ha^−1^) are not frequent in new plantations (Carmona-Torres et al., 2023). On the other hand, the most recent popular choice is the establishment of Super High-Density (SHD) plantations (780-2254 trees ha^−1^) mechanically pruned and harvested aiming at high yields with low cost per production unit (Díez et al., 2016). This planting system requires irrigation, trees with low vigor, high productivity, and suitability for mechanical harvesting (low fruit detachment force and low fruit injury rate) (Rallo et al., 2013). However, in the last decades, within hundreds of traditional cultivars only less than a dozen have been selected as appropriate for SHD plantations (Centeno et al., 2019; Olint, 2024).

The majority of traditional and the total of SHD olive groves are grown for olive oil production (IOC, 2024) and yields (amount of fruit and oil) are dependent on tree fruit load and fruit oil content (Lopez-Bernal et al., 2021). In addition, the genetic potential of olive cultivar, climatic conditions (air temperature, precipitation, solar radiation), and cultural practices (plantation design, irrigation, fertilization, pruning, pest management) are among the factors that define oil accumulation rate and the final output (Rosati et al., 2023). Using the human-based visual assessment of fruit color change is convenient for determining fruit maturity stage but it is prone to human subjectivity and error. Hence, to overcome such limitations RGB imaging can successfully be employed for the determination of maturity index (Ezenarro et al., 2023). However, RGB-based maturity index is not always coupled to oil content and it is a cultivar-dependent trait, requiring further research for predicting olive oil content from image processing (Navas-Lopez et al., 2019; Montanaro et al., 2023). This feature can be estimated with high accuracy using NIR spectroscopy devices (Lee et al., 2018) that are nowadays available in some olive mills too. However, destructive fruit sampling is required, not to mention the cost of purchasing and maintenance of the equipment. Hence, a fast, simple, non-destructive and field scale method for fruit oil content determination would be highly appreciated by the olive industry (Montanaro et al., 2023). In line with this, a single non-destructive maturity index based on the absorbance spectrum has been applied on single, intact olives of the Leccino cultivar (Cinosi et al., 2023). In recent years, great progress has been achieved in farm practice optimization using drones, multispectral and thermal cameras, and sensors (e.g., proximal, remote, optical, in-planta) mainly for decision-making in irrigation, fertilization, and pest management (Tsouros et al., 2019). However, their application in olive oil estimation is still limited. In addition, this digital approach provides large-scale data, collected fast and non-destructively but requires investment in equipment and technical skills. In contrast with this, simple Red, Green, and Blue (RGB) imaging has shown promising results within affordable plant phenotyping, combining low cost and ease of use and widely accessible, since this technology is even onboard in smartphones (Reynolds et al., 2019). Within a plant science context, RGB imaging provides valuable information on various traits including plant water status (Zakaluk and Ranjan, 2008), foliar disease classification and severity (Alves et al., 2022), and ripening monitoring via chlorophyll concentration estimation in annual (Luis Fernando et al., 2020) and perennial (Hoda et al., 2022) crops. Recently, RGB images have been employed to estimate some fruit quality traits such as oil and phenols in olive (Montanaro et al., 2023) and sugars content in grapevine (Wei et al., 2022). These findings showed the seasonal pattern of RGB is not linear causing a hysteretic correlation between RGB bands and the quality trait and in turn a looping behavior of the input-output plot *in sensu* Morris (2012).

The hysteresis phenomena is relatively common in electronics (e.g., Morris, 2012), geophysics (e.g., Paterson et al., 2018), and human health (e.g., Ross et al., 2016). Within plant science, some physiological responses to stimuli show a hysteresis such as the efficiency of photosynthetic apparatus triggered by light availability (Serôdio et al., 2022), the diurnal transpiration (Amato et al., 2021). The hysteresis phenomenon has been also reported in field studies in cherry and olive explaining the diurnal growth of fruit in response to *VPD* (Khosravi et al., 2021; Zucchini et al., 2021).

In addition, the pattern of leaf area index and RGB (satellite, drones) derived indexes might be hysteretic (Peichl et al., 2015; Gong et al., 2021).

However, the hysteresis issue within imaging of fruit quality was not adequately considered. The hysteretic pattern of olive fruit quality traits (oil, phenols) in response to R, G, and B pixel values has been previously recognized over two growing seasons (Montanaro et al., 2023) but did not receive so much attention. Similarly, in a study on *Vitis* spp. although the pattern of color bands subtended a hysteretic response of the analyzed trait, the hysteresis did not enter the discussion (Wei et al., 2022).

The non-linearity of a predictor (e.g., the seasonal RGB) is challenging within a modelling exercise. Hence, based on the ability of artificial neural networks (ANNs) to cope with nonlinear problems (Wei et al., 2022), RGB imaging and ANNs have been combined to determine key olive fruit quality traits (Ram et al., 2010; Montanaro et al., 2023).

The application of ANNs is increasing in various sectors including agriculture (Attri et al., 2024). However, ANNs not always provide a solution based on a mechanistic approach, and often require a pre-processing step to minimize embedded constraints such as overfitting (Bejani and Ghatee, 2021). In addition, a general linear model (GLM) may perform as well or even better than the ANN and will save time (Özesmi et al., 2006). Following this, additional models such as GLM are desirable to expand the set of available computational processes for predicting fruit quality traits from images.

Based on this background, this paper presents a protocol accounting for the hysteresis for the selection of RGB-based predictors to be used in GLM.

## 2 Materials and methods

### 2.1 Sampling and determination of oil concentration

Experiments were carried out during the 2023 season in olive groves located in Southern Italy (Metapontino area). Starting from the end of July, olive samples were collected every 7-10 days till the end of November. At each sampling point, ~300 g bulk samples (×3) were collected from super-high density (SHD, 1.5 × 4 m) groves of the 4-year-old cultivar Arbequina and Koroneiki, Fasola (8.5 × 9 m, ~70-year old), Frantoio (6 × 6 m, 20-year old), Leccino (6 × 6 m, 30-year old) and Maiatica (10 × 10 m, ~60-year old) plants. Olive groves were irrigated and managed according to local practices. Olive fruits were collected from the various sides of the canopy of 5-6 trees. After the imaging acquisition (described in detail in subsequent section), each fruit sample was ground (skin+flesh+stone) into a paste with a hammer mill, and about 75 g of a well-mixed subsample of paste was used for a single determination of extractable fat matter (oil, % fresh weight), and water content (% fresh weight) using OliviaTM instrument (FOSS, Hillerød, Denmark) (Montanaro et al., 2023).

### 2.2 Imaging and colorimetric indexes

Image acquisition and data extraction followed Montanaro et al. (2023). Briefly, each sample (×3 subsample) was placed on a blue background and pictured using a Nikon D5100 digital camera (16.9 Mpixels, AF-P DX Nikkor 18-55 mm, f/3.5-5.6 G VR, Nikon, Tokyo, Japan) and a X-Rite ColorChecker enclosed in a photo studio box. The box (Ombar Photography Light Box) was equipped with LED 5500K, 100 LEDs on top, and sheltered through a light diffuser to avoid direct illumination of samples. Each picture includes a variable number of olive fruit depending on cultivar and sampling time. Namely, the mean fruit number ( ± SE) for the initial and last sampling point was: 169.4 ± 5.61 and 97.2 ± 2.8 in Arbequina, 47.4 ± 3.3 and 36.8 ± 0.6 in Fasola, 97. ± 4.7 and 57.2 ± 1.7 in Frantoio, 195.2 ± 7.1 and 111.2 ± 4.7 in Koroneiki, 118.2 ± 6.5 and 71.2 ± 2 in Leccino, 81.8 ± 4.8 and 51.5 ± 1.4 in Maiatica. The olives were not rotated hence the one side of each fruit was pictured. A total of 245 images were collected as JPEG. Images were then segmented to remove the background using ImageJ 1.53t version (Schindelin et al., 2019), and the “measure RGB” plugin to measure the mean pixel values of Red (R), Green (G), and Blue (B) primary color. The primary colours were then recombined producing 35 colorimetric indexes (CI) (**Supplementary Table S1**).

### 2.3 Modelling oil concentration

The R, G, and B mean pixel values were used as predictors of the olive oil concentration (%FW) (*Y*) according to the following linear model:


(1) Y~Red+Green+Blue

After checking for collinearity employing the analysis of variance inflation factor (VIF) (Chatterjee and Simonoff, 2013), the model [1] was reduced removing the predictor(s) having the VIF>5 (Marcoulides and Raykov, 2019). Additional models, employing a colorimetric index (*CI*) as predictor, had the following general formula:


(2) Y~CI

Each *CI* candidate predictor (**Supplementary Table S1**) was selected based on the *Fitness Index* (*FI*) determined as:


(3) FI=abs(Hyst)+abs(ρ)

where *Hyst* represents the hysteresis index (see Equation 4) and ρ the Spearman’s rank correlation test calculated over the *Y* and *CI* pairs. The values of *Hyst* were calculated according to (Kosmulski et al., 2009 and Serôdio et al., 2022) based on the normalized difference between the upward (increasing *CI* values) and downward (decreasing *CI*) phases of the hysteresis:


(4) Hyst = (hyst_area–(Tot_area–hyst_area))/Tot_area

The *hyst_area* represents the area under the reverse harm of the curve fitting the *Y* (oil concentration) *vs CI* scatter of the points occurring after the maximum *CI* (*CI_max*) was detected (i.e., decreasing *CI*). The *Tot_area* is the area under the curve fitting all the *Y*, *CI* pairs and was calculated assuming a rotation of the “hysteretic” harm attributing to the original decreasing *CI* point a new synthetic *CI* value (*CI_syn*) based on their original value (*CI_orig*) and *CI_max*:


(5) CI_syn=CI_max+(CI_max–CI_orig)

**Figure 1:**
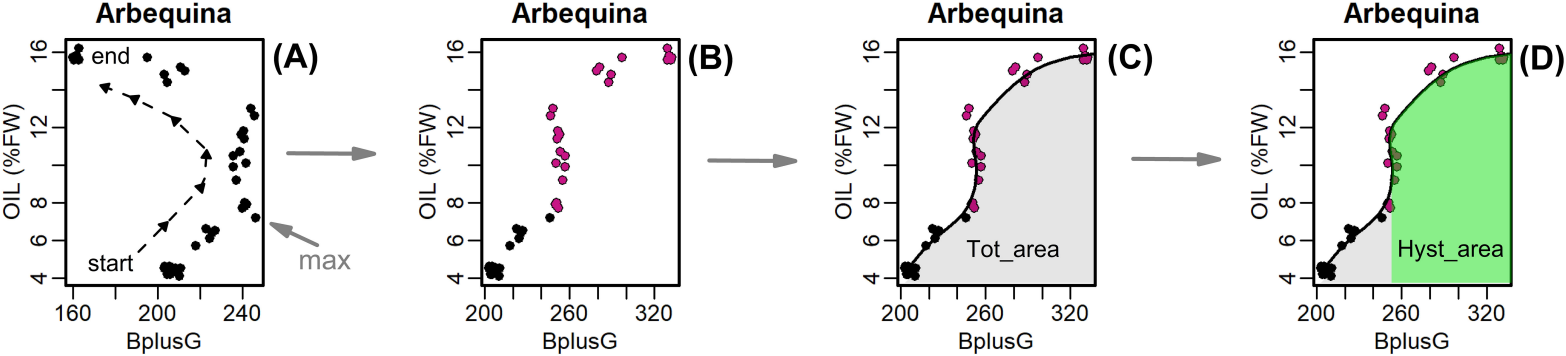
Example of the process for the determination of the arguments of the *Hyst* index function (Equation 4) employing the seasonal dataset of the cultivar Arbequina and the BpluG colorimetric index. In **(A)** it is reported the original scatterplot of the correlation between oil concentration (%FW) and the BplusG colorimetric index, the dashed line indicates the time direction (start → end) of the season, the maximum value of the BplusG is indicated by the arrow and was considered the beginning of the reversing direction (hysteresis harm); **(B)** the points occurring after the BpluG max, were identified (purple dots) and plotted assuming no hysteresis; **(C)** the whole points were fitted (continuous line) determining the area under the curve (AUC) which was labeled as *Tot_area*; **(D)** the portion of the AUC belonging the hysteretic (purple) points was determined and labeled as *Hyst_area*.

Hence, the original decreasing *CI* values detected after the max *CI* was reached, were rotated having a synthetic increasing *CI* value. As an example, using data of the cultivar Arbequina and BplusG colorimetric index the **Figure 1** visualizes how the various components of Hyst (Equation 4) have been determined. According to Serôdio et al. (2022), the values of *Hyst* ranged from 1 to -1, with negative values corresponding to negative hysteresis (clockwise hysteresis) and positive values corresponding to positive hysteresis (counterclockwise or hysteresis; downward phase higher than upward phase).

The selection of the *CI* to be evaluated as predictor was than based on their *Fitness Index* (Equation 3). Namely the *CI* having a *FI* falling in the top 1^st^ quintile were selected as predictor (see Equation 2).

Each model (*m*) in Equations 1 and 2 was tested within each cultivar and its performance appraised by the coefficient of determination (*R* ^2^), the root mean squared error (RMSE), and the mean absolute error (MAE) which were then combined in the Global Performance Indicator (GPI) (Montanaro et al., 2023 and references therein). Briefly, the GPI was determined considering the [0, 1] normalized values of *R* ^2^, RMSE, and MAE (*i*):


(6) $$GPI_{m}=\sum_{j=1}^{5}\;\;\alpha ({\bar{O}}_{i}-O_{ij})\;$$

where *α* equals -1 for i = *R* ^2^ and 1 for RMSE and MAE, *O_i_* and ${\bar{O}}_{i}$ were the value and the median, respectively. The median was calculated over the five iterations (*j*) (see below). After the *GPI* were determined, considering that “the more the accuracy of the model the higher the value of the GPI” (Despotovic et al., 2015), the models were ranked according to GPI.

All the dataset in each cultivar (45, 40, 40, 45, 45, 30 in Arbequina, Fasola, Frantoio, Koroneiki, Leccino and Maiatica, respectively) was randomly split into training (70%) and testing (30%). The training was used for model parametrization (minimizing the squared residuals) between RGB-based CI (covariate) and olive oil concentration (response variable). The same training and testing datasets were used as benchmark dataset across the various models preventing any stochastic effect due to random splitting. In addition, to account for the randomness of the training (and testing) dataset, this random subsampling was repeated five times according to Montanaro et al. (2023).

### 2.4 Statistical analysis

A one-way ANOVA was used to examine the differences between treatments after checking the hypothesis of normality (Shapiro-Wilk’s test) and equal variance (Levene’s test) tested at *p =* 0.05. Following the failure of the test of the ANOVA assumptions, the nonparametric Kruskal–Wallis’ test was employed. The residuals of each single model were calculated as the difference between observed and fitted values. Residuals from the five iterations were pooled before the analysis which was conducted to evaluate their randomness and constancy of variance. All data analysis and plotting were conducted in R (R Core Team, 2021).

## 3 Results

### 3.1 Seasonal variation in oil concentration and R, G, B

**Figure 2:**
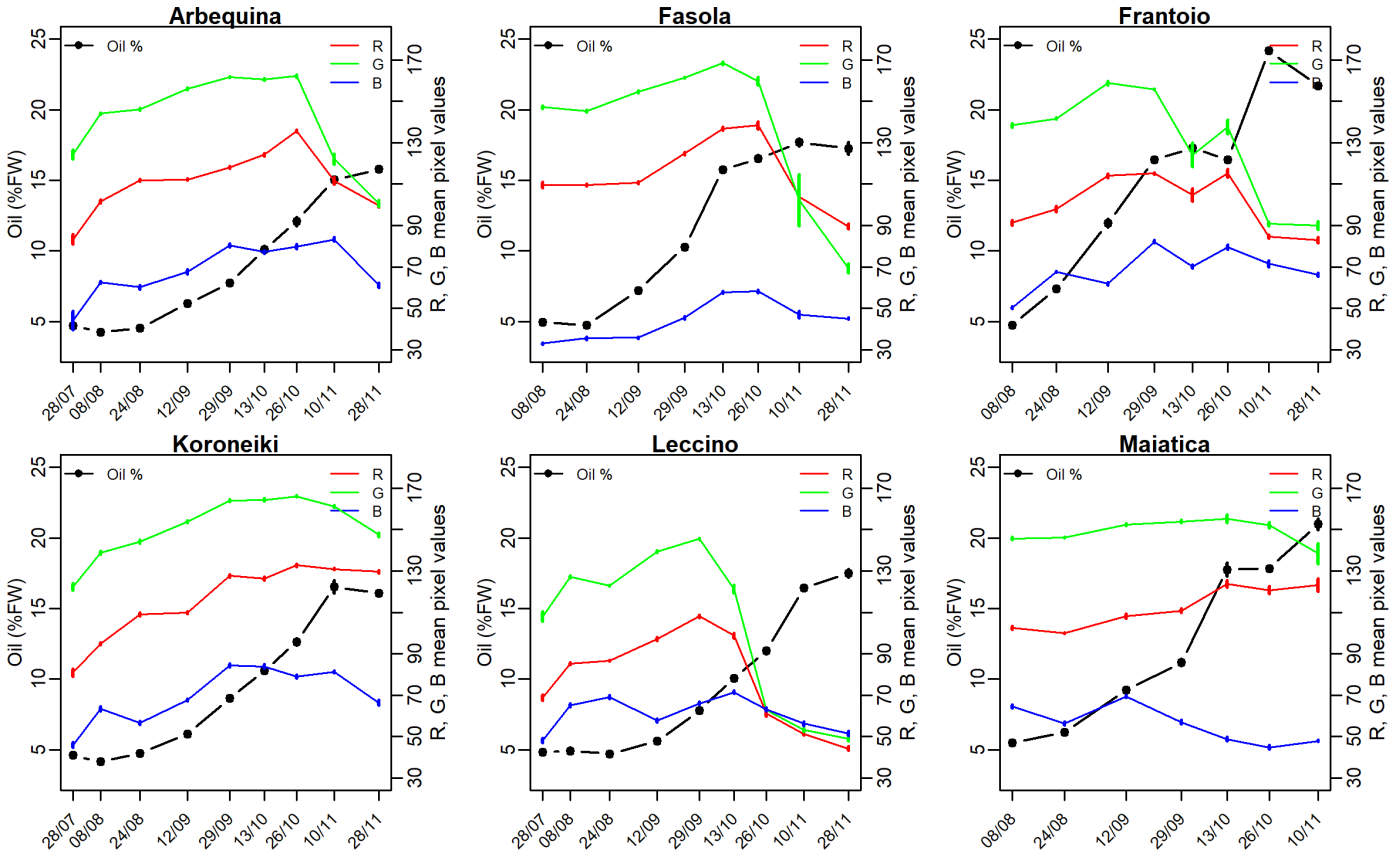
Values of the mean oil (%FW) (•) and Red (R), Green (G), and Blue (B) mean pixel values recorded throughout the season in olive cultivars from traditional (Fasola, Frantoio, Leccino, Maiatica) and super-high density (Arbequina, Koroneiki) plantation systems. Vertical bars are standard error and are visible when larger than symbol or line.

Oil concentration linearly increased in all cultivars from ~ 5% FW recorded at the end of July up to an asymptotic value of ~ 15.5% (Arbequina, Koroneiki, Fasola, Leccino) recorded on November 11^th^ and earlier (October 10^th^) in Fasola cultivar (**Figure 2**). It is worth noting that the final oil concentration approached ~ 20% and ~ 24% in Maiatica and Frantoio, respectively (**Figure 2**). Fruit development (fresh weigth per single olive fruit) and water content (%) are reported in **Supplementary Figure S1**. The R, G, and B mean pixel values showed a nonlinear pattern throughout the experiment in all cultivars with an initial increasing trend followed by a decreasing one. However, in Maiatica the G color band was stable at about 150 mean pixel value for almost the whole season and declined only at the last measuring point (**Figure 2**). The R, and G color bands abruptly declined by the end of October in Fasola and Arbequina, while such a change was recorded about one month earlier in Frantoio and Leccino (**Figure 2**), early in the season (end of September) (**Figure 2**). In addition, the pattern of the R mean pixel value in Koroneiki and Maiatica missed the late season declining part (**Figure 2**). As concerning the trend of G color band in Koroneiki and Maiatica, it showed the less evident seasonal variation compared to that in the other cultivars (**Figure 2**). Particularly, in Maiatica the G had a narrow sesonal range (from ~ 116 to ~ 125). Generally, the B color band in all cultivars showed a less pronounced declining pattern compared to that of R and G. In addition, in Maiatica the B color band start soon to decline if a transient increase at about mid Septemeber is exluded. **Figure 2** also shows the similarity of B trend in Arbequina and Koroneiki.

**Figure 3:**
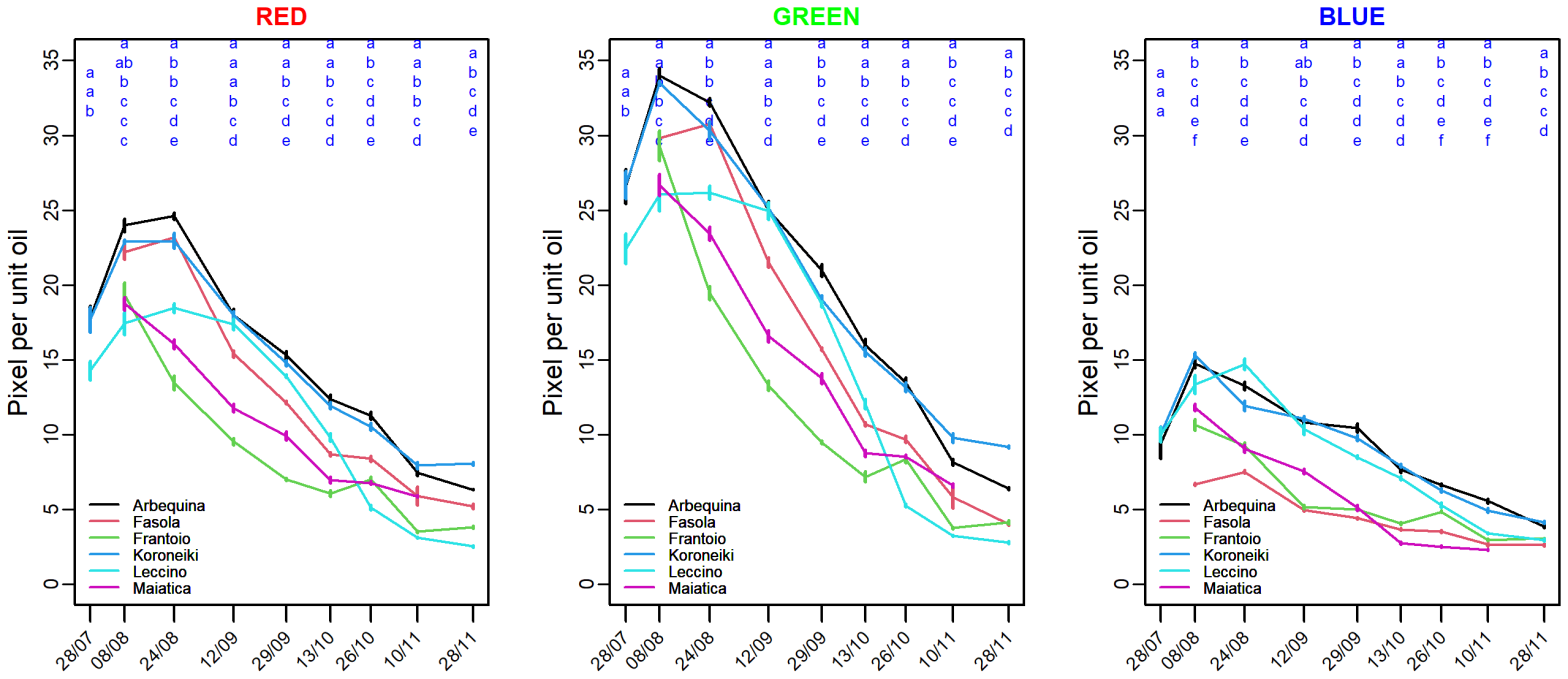
Mean (left) Red, (middle) Green, and (right) Blue pixel value normalized per unit of fruit oil concentration (%FW) recorded in various olive cultivars grown in super-high density (Arbequina, Koroneiki) and traditional plantation systems (Fasola, Frantolio, Leccino, Maiatica). Comparing cultivars within the same sampling time, the different letter indicates significant differences (Kruskal–Wallis’ test, *p*< 0.05).

**Figure 3** reports the seasonal R, G, and B mean pixel values normalized per unit of oil concentration. It can be noted a similar declining pattern across the examined cultivars and color bands, moreover an initial ~ 45% increase in R, G, and B values is shown at least for those cultivars sampled also at the end of July (i.e., Arbequina Koroneiki, Leccino) (**Figure 3**). The SHD cultivars (Arbequina and Koroneiki) showed the higher pixel per unit oil compared to the other cultivars consistently in most of the sampling occasions, in addition the normalized R and G in these SHD cultivars remain comparable in about 60% of sampling times (**Figure 3**). In addition, a temporal shift of the R, G, and B normalized values can be envisaged across cultivars, particularly in Leccino that normalized value start to decline 2-3 weeks late (**Figure 3**).

**Figure 4:**
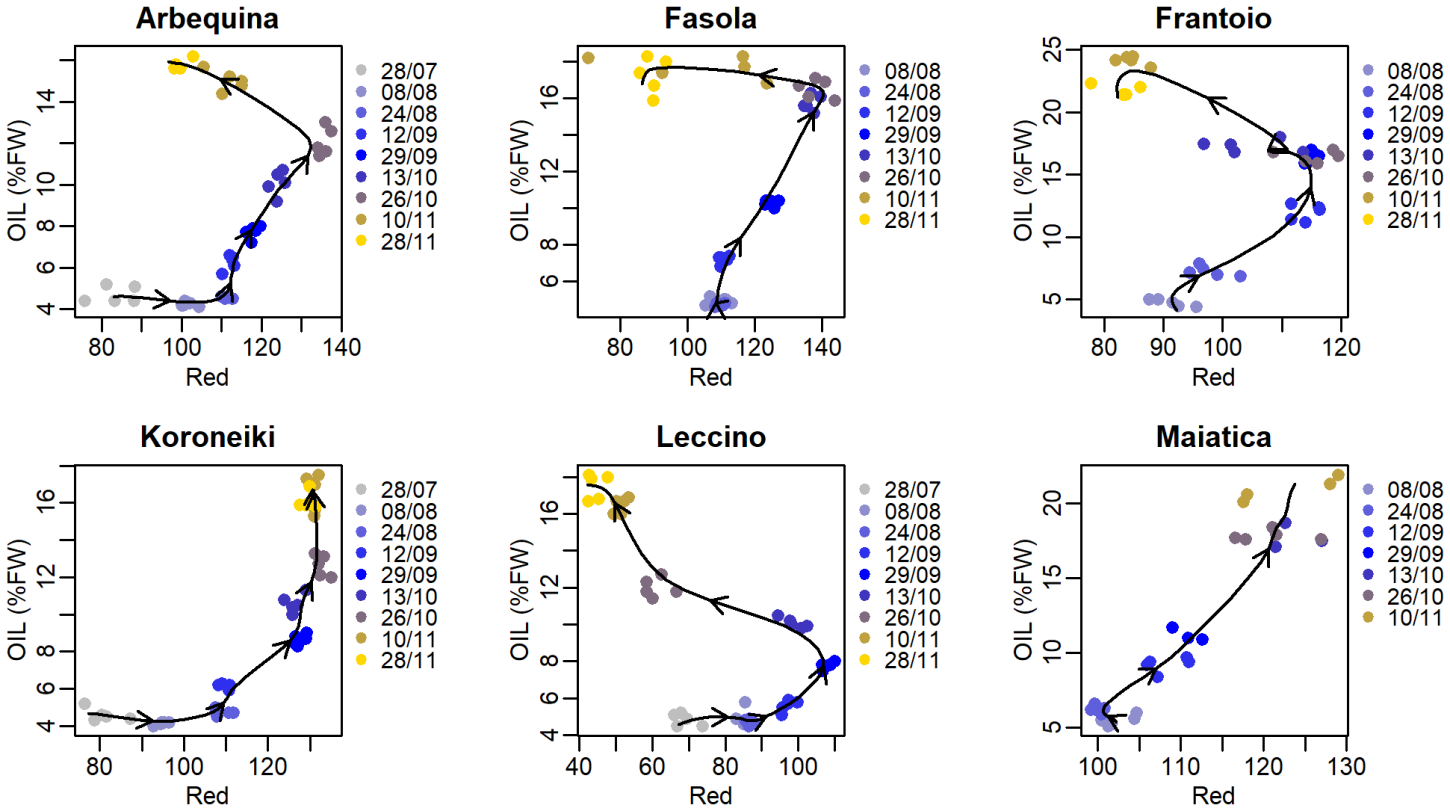
Correlation between mean pixel values of the Red color band and oil concentration (%FW) in various olive cultivars. In each cultivar, data from all sampling times were grouped before fitting (cubic smoothing spline), and arrows over the fitting line indicate time direction.

**Figure 5:**
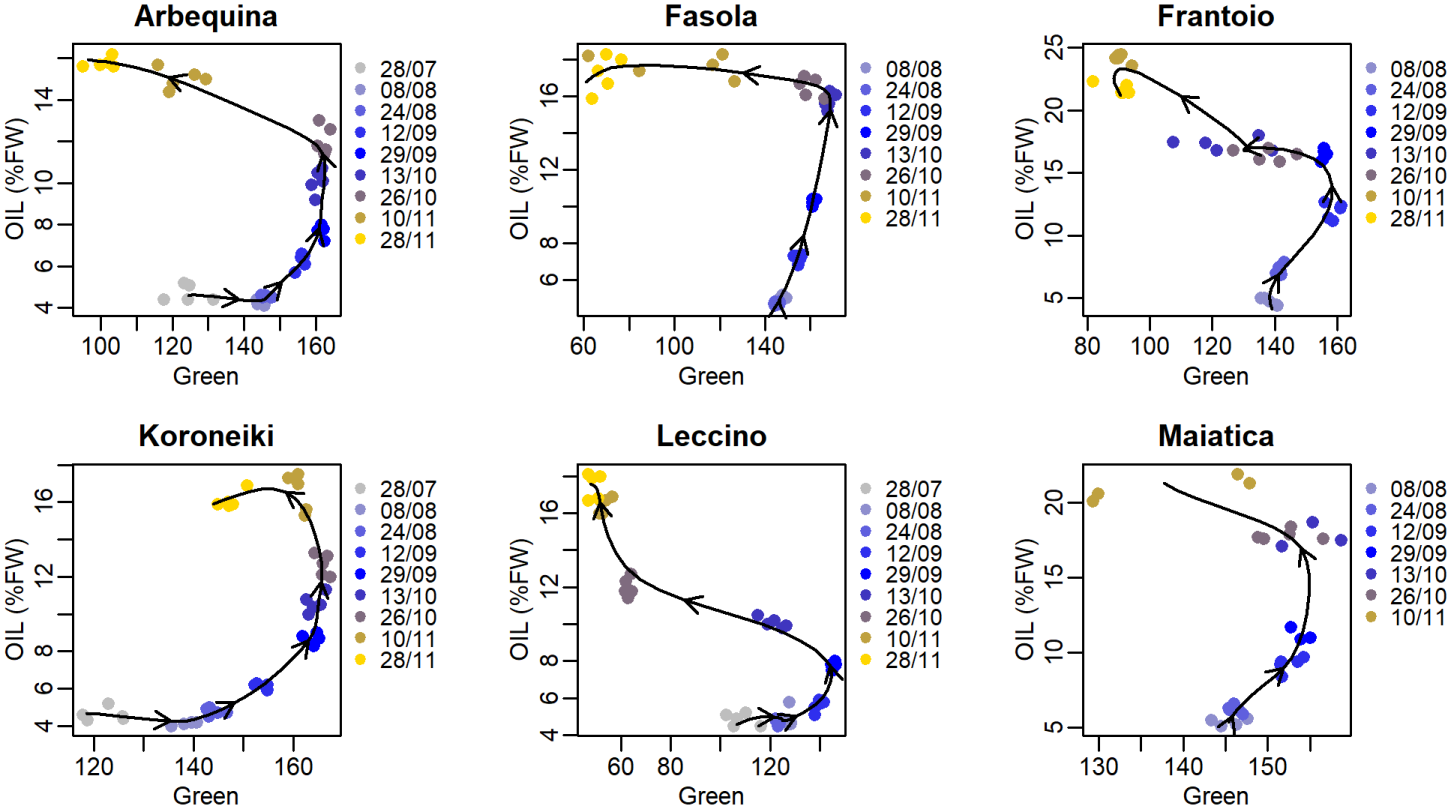
Correlation between mean pixel values of the Green color band and oil concentration (%FW) in various olive cultivars. In each cultivar, data from all sampling times were grouped before fitting (cubic smoothing spline), and arrows over the fitting line indicate time direction.

**Figure 6:**
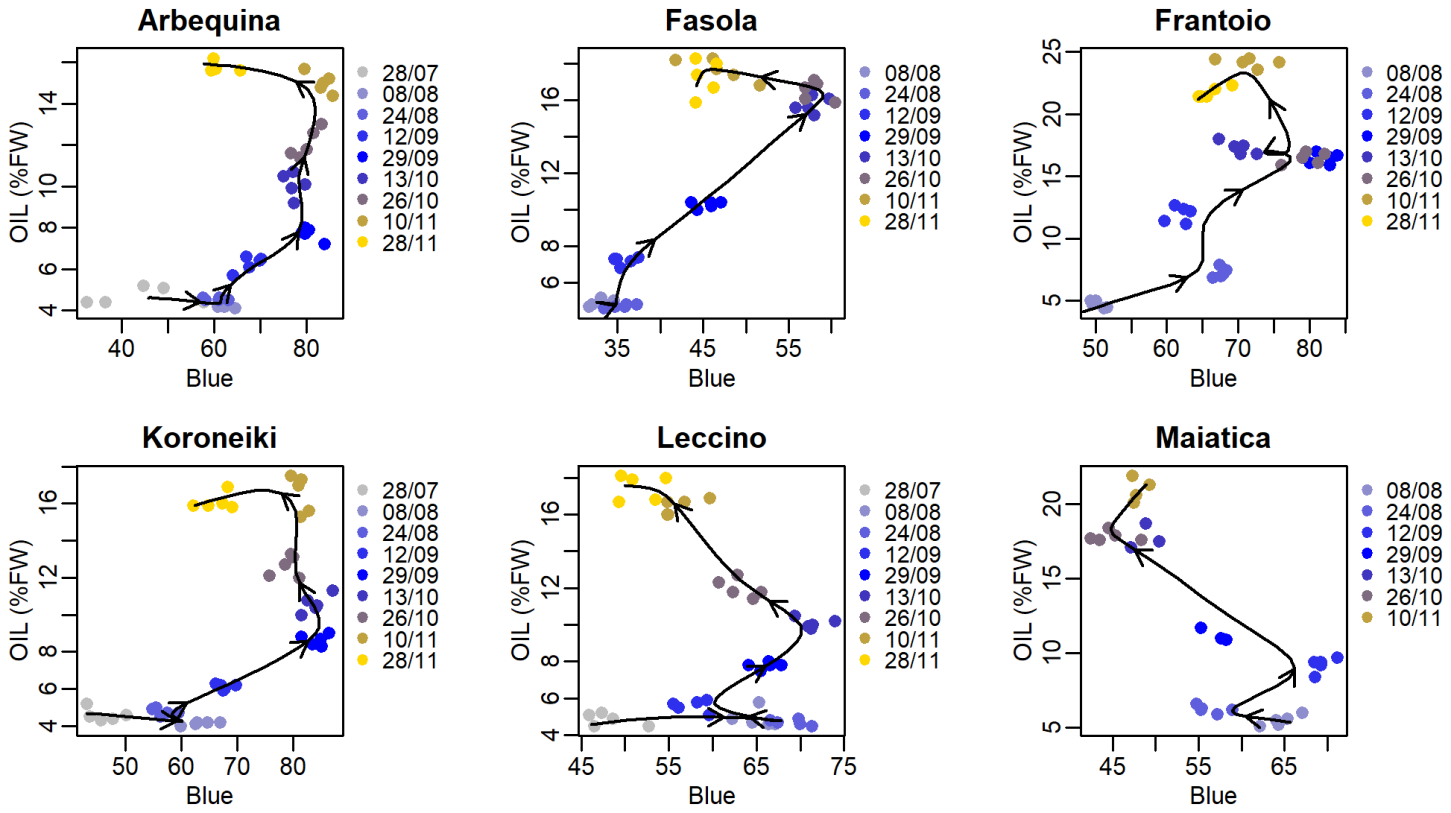
Correlation between mean pixel values of the Blue color band and oil concentration (%FW) in various olive cultivars. In each cultivar, data from all sampling times were grouped before fitting (cubic smoothing spline), and arrows over the fitting line indicate time direction.

Correlation between the seasonal R, G, and B color bands and oil concentration showed a nonlinear hysteretic pattern (**Figures 4**–**6**). In contrast with this, the trend of the R color band in Koroneiki and Maiatica (**Figure 4**) does not show hysteresis. The timing of the onset of the hysteresis differed across cultivars and color bands. However, it was consistently advanced in Leccino (end of August, beginning of September) compared to that recorded in all other cultivars (end of October).

**Figure 7:**
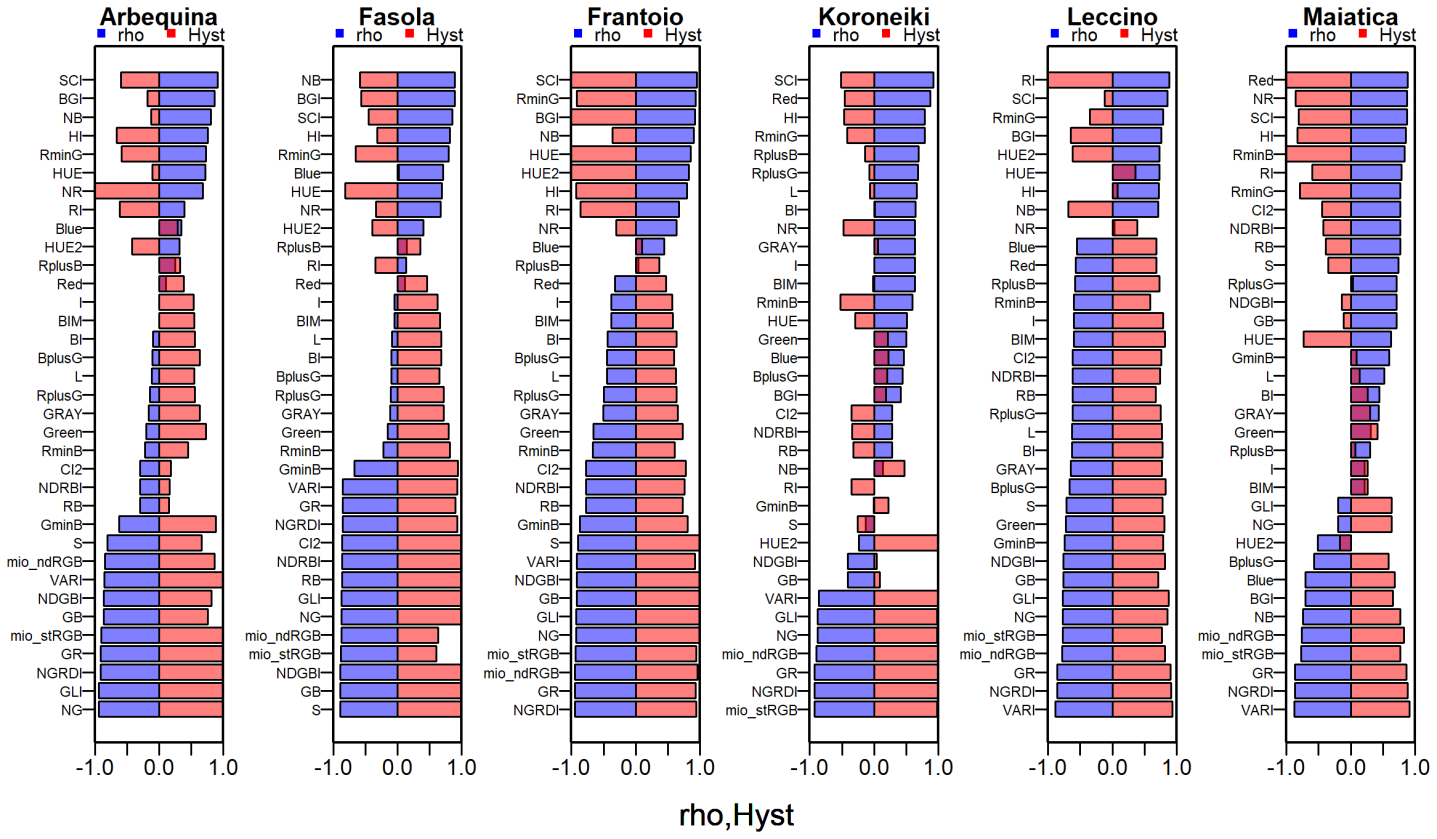
Values of the Spearman’s coefficient (rho) of the correlation between mean pixel values of the Red, Green Blue color band or other RGB-derived indexes and oil concentration (%FW) and of the relative hysteresis area (Hyst) in various olive cultivars.

Values of Spearman’s correlation coefficient (ρ) determined over the abovementioned correlation between oil concentration and R, G, and B color bands and all the other RGB-based *CIs* (**Figures 4**, **6**) are reported in **Figure 7**. In parallel with the ρ, the relative hysteresis area in each cultivar was determined and reported in **Figure 7**, too.

**Figure 8:**
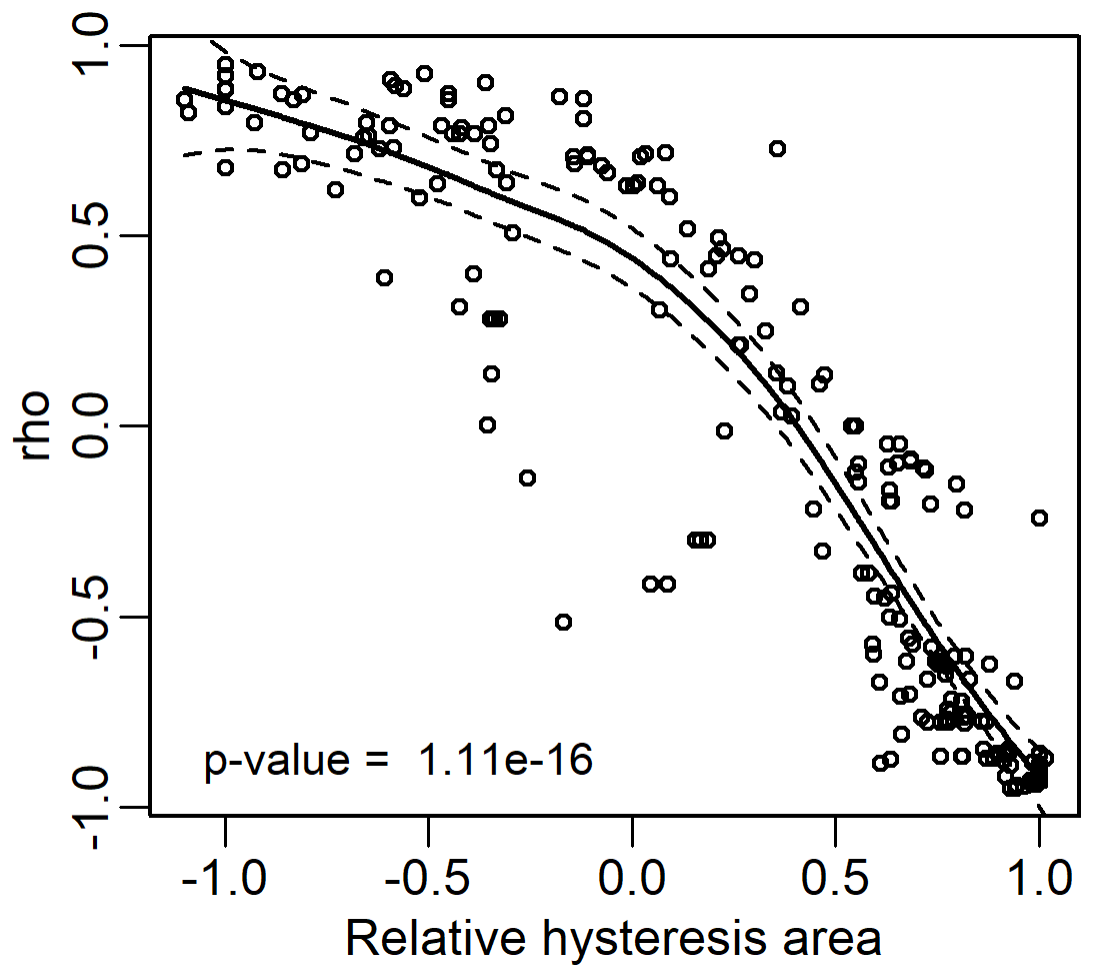
Scatterplot between the relative hysteresis area determined through the Equation 4 and the Spearman’s rank correlation test (ρ, rho) calculated over the correlation between each of the 35 CIs and oil concentrations in all cultivars. The continuous fitting line represents a general additive model, the whole data (*n*= 210) were pooled before fitting, dashed lines represent the 95% confidence bands.

**Figure 9:**
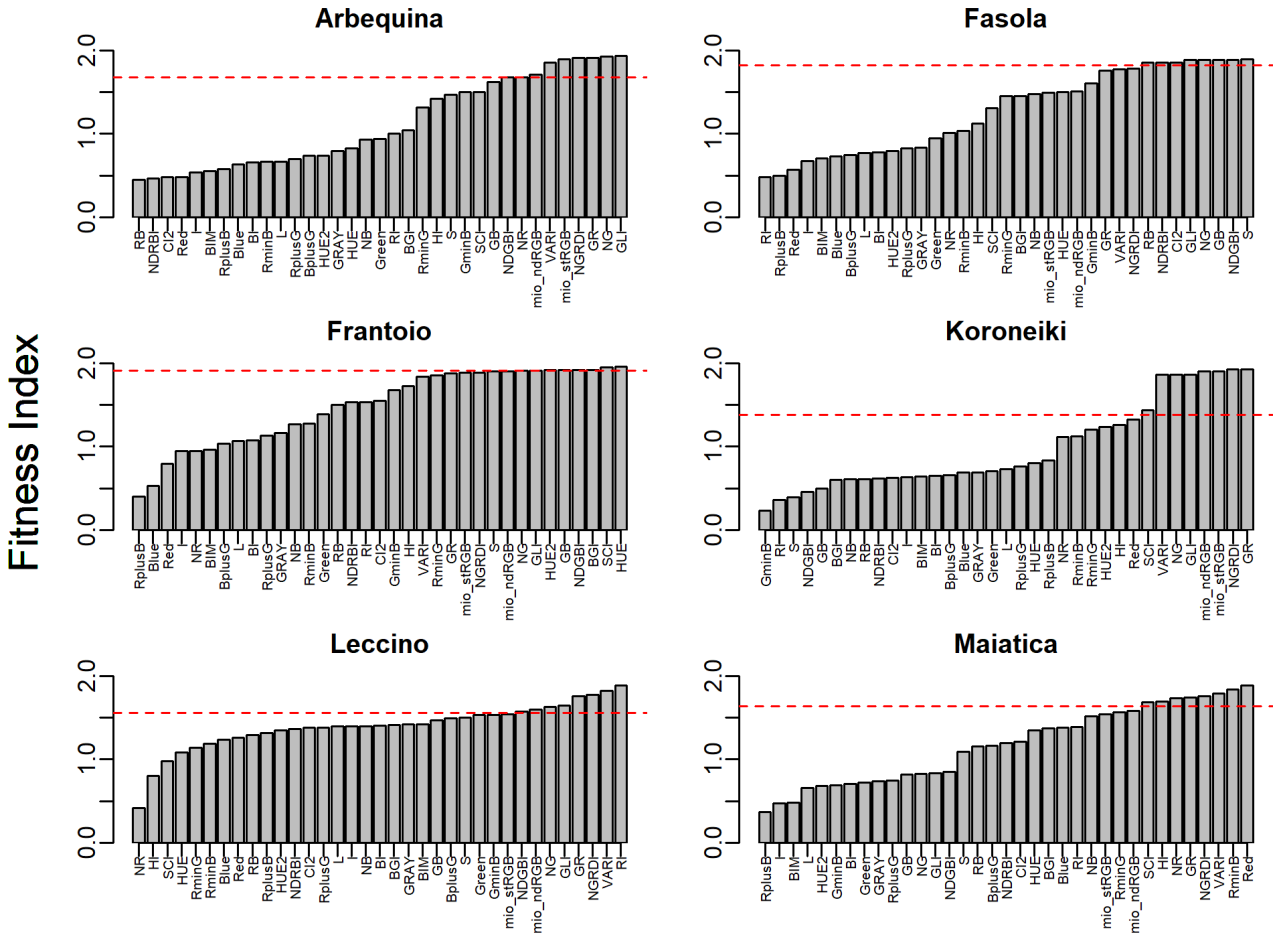
Variation of the Fitness Index (*FI*, Equation 2) of the various colorimetric indexes and color bands to serve as predictors of the olive oil in Arbequina, Fasola, Frantoio, Koroneiki, Leccino, and Maiatica cultivars. The dashed horizontal lines limit the *FI* of the 1^st^ quintile of the *CIs*.

Focusing on the highest ρ values in Arbequina, 10 *CIs* showed a ρ ranging from -0.85 to -0.94, and in the other three ones it ranged from 0.81 to 0.91. For the other cultivars, the number of *CIs* falling in a similar range was 17 in Fasola and Frantoio, while it was nine in Koroneiki and Maiatica and five in Leccino. In addition, **Figure 7** shows a good correspondence between ρ and *Hyst* across the *CIs* and color bands. In line with this, pooling the whole seasonal datasets across all cultivars, the correlation between Hyst and ρ showing that ρ values closest to 1 or -1 corresponded to values with no (*Hyst* = -1) or early hysteresis (*Hyst* close to 1) (**Figure 8**). The **Figure 9** reports the Fitness Index (*FI*) which combines the relative hysteresis area (*Hyst* index) and ρ determined over the correlation between each of the 35 *CIs* and oil concentration. The first quintile of *CIs* or color bands, i.e. the 20% of *CIs* with the highest *FI*, (see red dotted line in **Figure 9**) had the following *FI* range: 1.69:1.93 (Arbequina), 1.86:1.89 (Fasola), 1.91: 1.96 (Frantoio), 1.44:1.93 (Koroneiki), 1.58:1.89 (Leccino), and 1.68:1.88 (Maiatica). Interestingly, Arbequina and Koroneiki cultivars share several common *CIs* falling in that range (“NGRDI”, “VARI”, “mio_stRGB” “mio_ndRGB”, “NG”, “GR”, “GLI”). Similarly, Fasola and Frantoio had common CIs (“NG”, “GB”,”GLI”,”NDGBI”) and Leccino and Maiatica (“NGRDI”, “VARI”, “GR”).

### 3.2 Image-based olive oil predicting models

**Figure 10:**
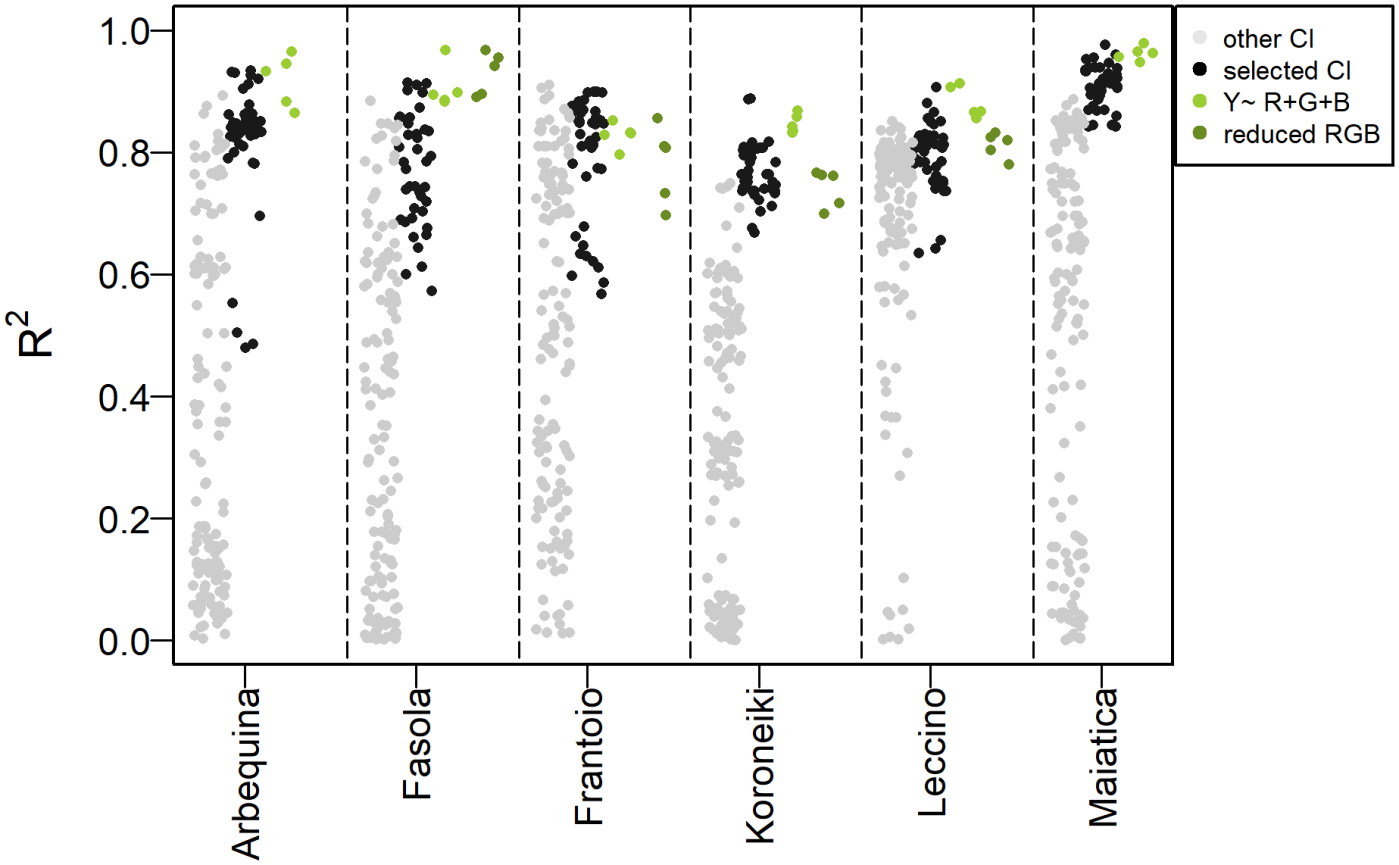
Distribution of *R* ^2^ values of the *predicted ~ true* oil (%FW) correlation achieved after the five iterations of the model *Y’*~ *CI* (Equation 2) using the “selected” and all the other colorimetric indexes (“other CI”), and the *Y*~ *R+G+B* model and the reduced version (*Y*~ *G+B* for Fasola, Frantoio, Leccino and *Y*~ *R+B* for Koroneiki). Note that each model type is plotted next to each other within the same cultivar separated by the dashed line and that the “selected” CI models were those identified based on the first quintile distribution of the fitness index (Equation 3, **Figure 9**).

The selection of predictors employed in olive oil modeling was based on *FI* (combination of ρ and relative hysteresis area). These predictors generated models with a median accuracy (*R* ^2^) of 0.80-0.84 in Arbequina, Frantoio, Leccino, of about 0.77 in Fasola and Koroneiki, of 0.91 in Maiatica. (**Figure 10**). Similarly, combining Red, Green, and Blue in a single linear model (*Y~ Red + Green + Blue*) was effective in predicting the olive oil concentration with a median *R* ^2^ values about 0.90-0.96 in Arbequina, Fasola, and Maiatica, of about 0.83-0.87 in Frantoio, Koroneiki, and Leccino (**Figure 10**).

**Figure 11:**
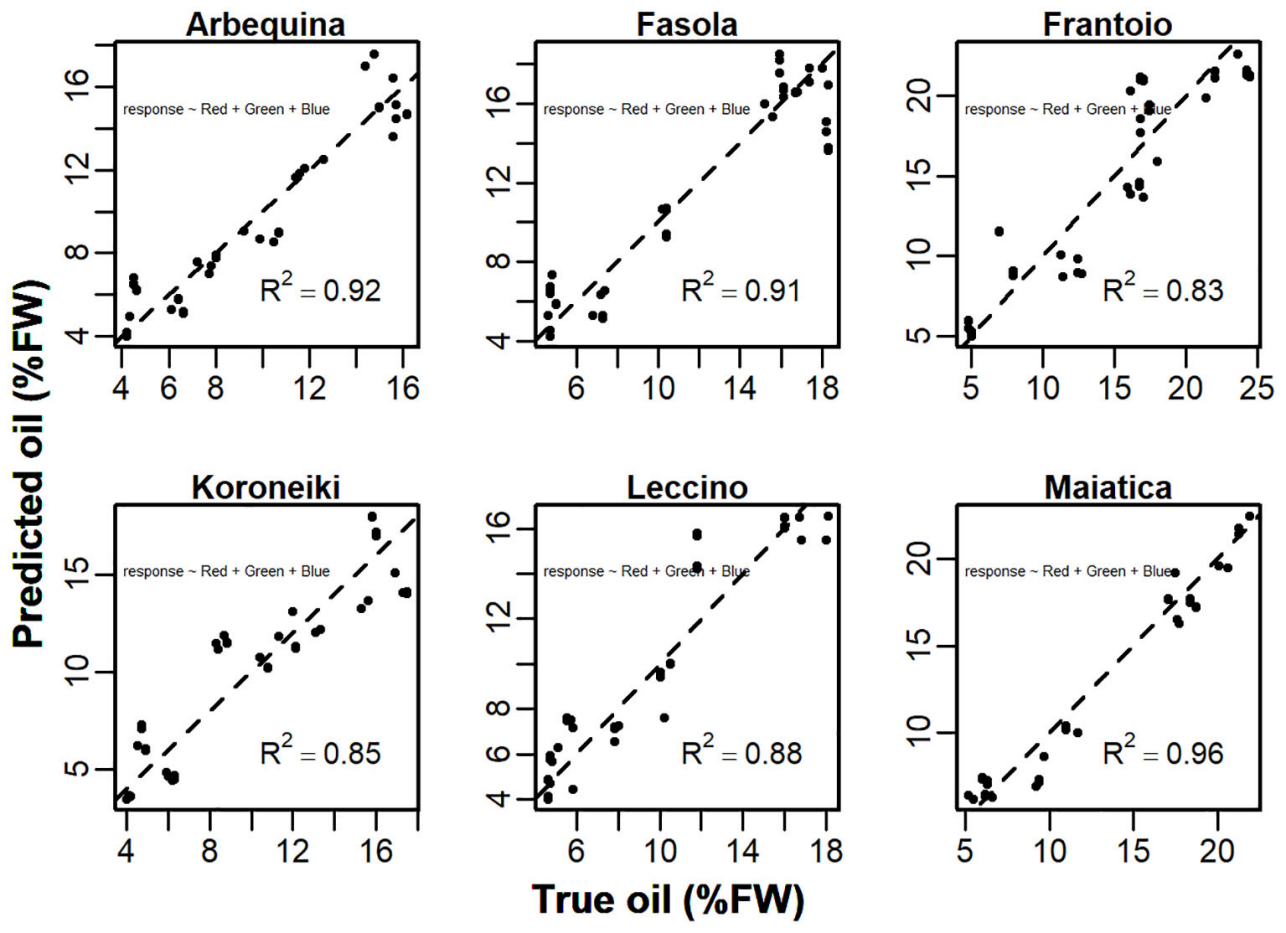
Scatterplot of the *predicted ~ true* oil (%FW) correlation of the model *Y*~ *Red+Green+Blue* (Equation 1). Values of the *R* ^2^ are the mean determined over the five iterations. The dashed line represents the 1:1 straight line.

**Figure 12:**
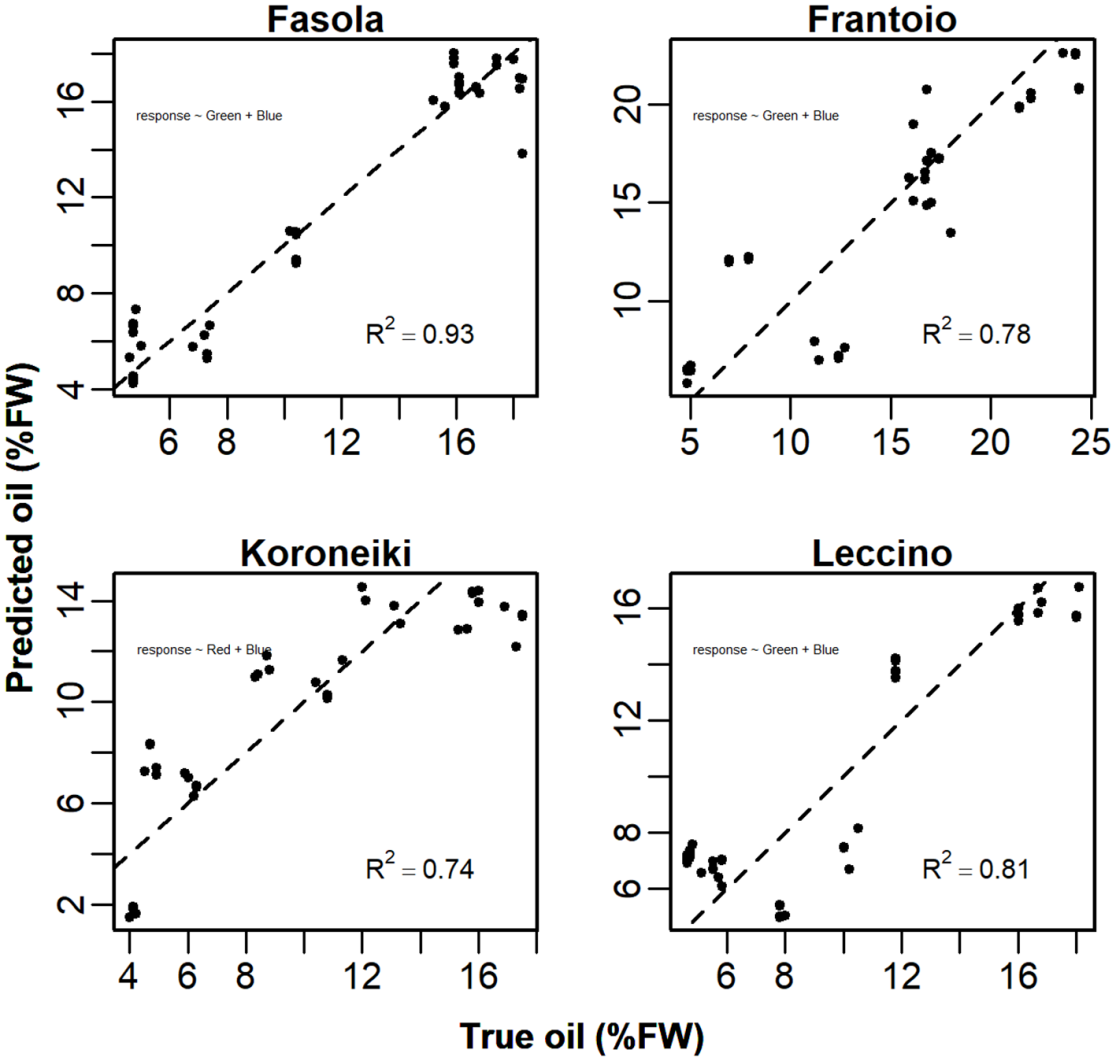
Scatterplot of the *predicted ~ true* oil (%FW) correlation achieved using the reduced model *Y*~ *Green+Blue* (Fasola, Frantoio, Leccino) and *Y*~ *Red+Blue* (Koroneiki) after the VIF analysis (**Tables 1** and **2**). The dashed line represents the 1:1 straight line.

**Figure 13:**
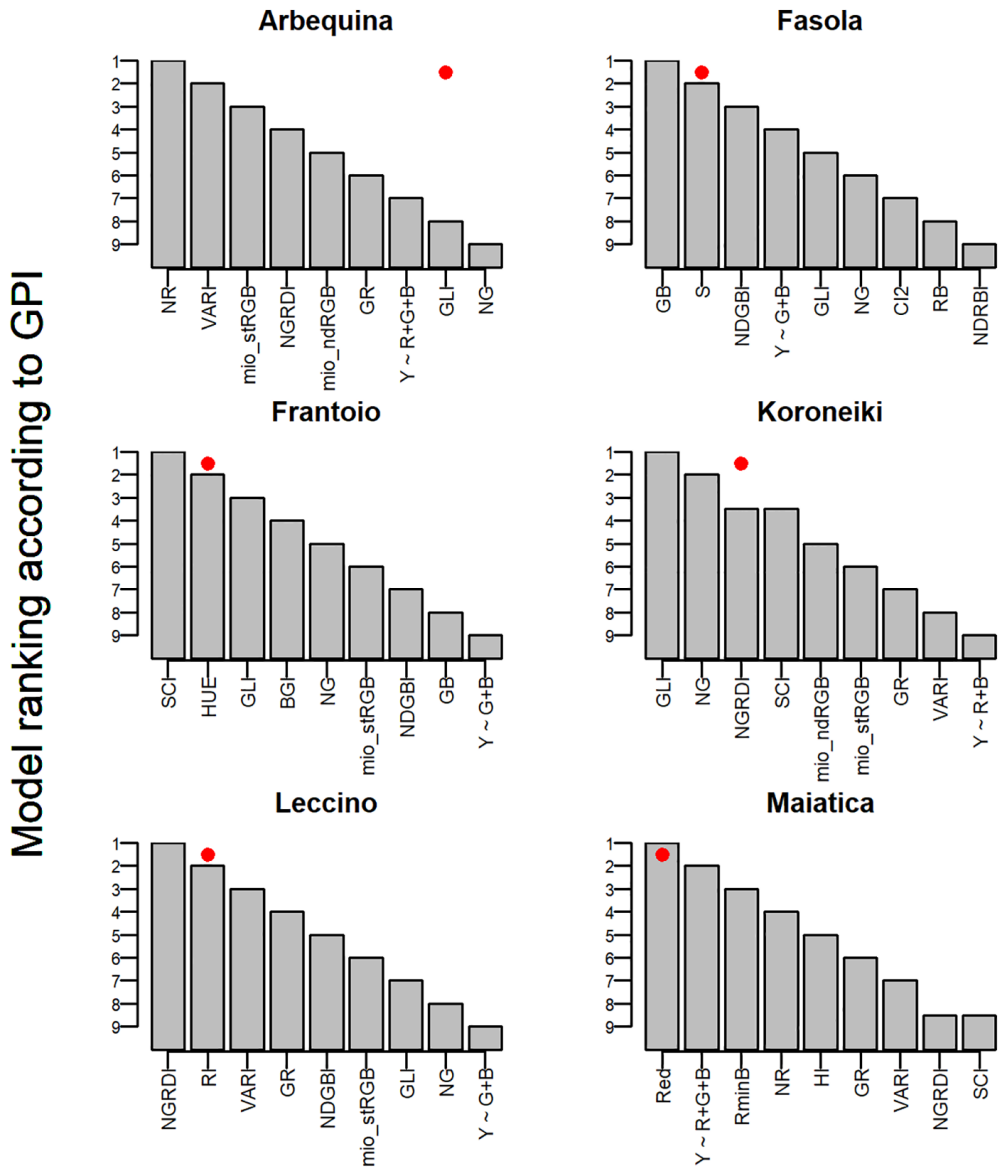
Ranking according to *Global Performance Indicator* (GPI) of the RGB-based additive linear models (*Y*~ *R*+*G+B*) or of the reduced version (*Y*~ *G+B*, *Y*~ *R+B*), or employing a single color band (Red), and of the *Y*~ *CI* (top quintile selected colorimetric indexes **Figure 9**) in olive cultivar from traditional (Fasola, Frantoio, Leccino, Maiatica) and super-high density (Arbequina, Koroneiki) plantations. In each panel, the red dot indicates the colorimetric index with the highest Fitness Index (**Figure 9**).

Detailed scatter plots between *predicted:true* oil show that the model *Y~ Red + Green + Blue* was able to explain about 83-96% of the total variance (**Figure 11**). However, the analysis of VIF revealed the collinearity (VIF>5) between some predictors in Fasola, Frantoio, Koroneiki, and Leccino (**Table 1**). Hence, for these cultivars a reduced model was then employed discharging the predictor with the highest VIF at the cost of reducing the *R* ^2^, except for the Fasola (**Figure 12**). All the scatterplots of the *predicted ~ true* olive oil correlation employing as predictor the CI or color band selected using the *FI* are reported in **Supplementary Figures S2:S7** along with their residuals (**Supplementary Figures S8:S13**). The overall accuracy of each predicting model was then appraised by the GPI collating the results of *R* ^2^, MAE, and RMSE (**Figure 13**). The GPIs were sorted in ascending order within the same cultivar, facilitating the interpretation of results considering that the higher GPI the better is the overall accuracy of the model.

## 4 Discussion

This study reports on seasonal oil accumulation in olive cultivars grown under traditional and SHD plantations and its image-based linear modeling contributing to progress towards digital olive crop science. In olive crop, there is a seasonal change in oil concentration and other quality traits (e.g., phenolic compounds, Roshani et al., 2016), hence information on the occurrence of these traits is pivotal to schedule the harvest time according to the entrepreneur’s target. For this purpose, there is an increasing development of crop management tools including those based on imaging assisting in fast and reliable monitoring of olive fruit traits (Gracia and León, 2011; Bellincontro et al., 2013). Here, a common RGB sensor was employed to predict the seasonal oil (%FW) in fruit. These results were translated in oil per unit of R, G, and B mean pixel values (**Figure 3**) highlighting the effect of cultivar on that normalized value. Results are challenging to compare because of limited literature existing on this specific point. However, such cultivar-dependency of olive oil yield on RGB color fits with López-Huertas et al. (2021) who showed that the amount of olive oil extracted from 12 cultivars differed even though they had the same color class determined through the popular destructive Jaén method. Following this finding, results pointed out that the weight of R, G, and B as model predictors would differ according to the cultivar and that a genetic component should be considered in model architecture. However, this remains to be specifically tested.

The SHD cultivars examined are commonly cropped at a global scale (Díez et al., 2016) increasing the significance of this study. Similarly, the traditional cultivars included globally cultivated ones (i.e., Leccino and Frantoio) (Barranco et al., 2000). The seasonal variation in oil is consistent with the literature in both SHD and traditional cultivars (Lopez-Bernal et al., 2021) highlighting a sigmoidal pattern in all cultivars. However, the asymptote of that curve was reached earlier in Leccino confirming it is an early ripening variety (Barranco et al., 2000).

The development of image-based models designed to predict fruit quality traits (e.g., oil concentration) is the subject of recent increasing interest within a digital agricultural domain which often employs artificial neural networks (ANNs) (Montanaro et al., 2023; Ebrahimi et al., 2024; Yang et al., 2024). However, ANNs remain still a sort of “black box” with a poor mechanistic approach in selecting the predictors, even though it is powerful in minimizing the error of the prediction including in nonlinear problems (Nagesh et al., 2024). In addition, ANNs might require a high level of computational efforts and a pre-process of the predictors but sometimes performing similarly to a GLM to the extent that a comparison between ANN and GLM is recommended (Özesmi et al., 2006; McQuisten and Peek, 2009). Following this, the present GLM-based olive oil predicting model might integrate existing ones which are mainly based on ANNs (Ram et al., 2010; Montanaro et al., 2023).

In this study, the hysteretic (nonlinear) relationship existing between olive oil and R, G, and B color bands and other RGB-derived colorimetric indexes was pointed out. Generally, hysteresis is triggered by the history of the value of a response variable in relation to previous values of the input variable and it is a recognised problem in electronics (e.g., Morris, 2012), geophysics (e.g., Paterson et al., 2018), and human health (e.g., Ross et al., 2016). In addition, in plant science research several studies are recognizing hysteretic behavior of variables responding to stimuli. For example, examining the efficiency of photosynthetic apparatus in response to light availability (Serôdio et al., 2022), the diurnal signal sourced by organic sensors tracking sap mineral concentration in response to daily course of transpiration (Amato et al., 2021), and the response of soil CO_2_ emission to temperature (Dusza et al., 2020). In addition, the hysteresis is reported for the behavior of leaf area index and RGB (satellite, drones) derived indexes in rice and boreal mires (Peichl et al., 2015; Gong et al., 2021). However, in fruit quality science and imaging, hysteresis has not been adequately considered (Wei et al., 2022; Montanaro et al., 2023).

Hence, here a protocol was developed for the selection of image-based predictors to be used in linear models and accounting for the hysteresis. In a formal hysteresis, the loop of the variable tends to be closed meaning that the last point overlaps the initial one (Morris, 2012). In Khosravi et al. (2022), to characterize the shape of the diurnal hysteresis loop of the olive fruit diameter and *VPD* three definitions were suggested “partial, incomplete, and complete”. In the present study, the hysteretic loop of the oil concentration was incomplete. To explain this observation, it should be considered that the olive oil final accumulation value (ending point of the hysteresis) is always greater than the initial one due to biological processes (Lopez-Bernal et al., 2021). A similar not-closed hysteresis was also recognized analyzing the time dependence of the light response of photosynthesis in algae (Serôdio et al., 2022) and of methane flux in the alpine meadow (Chen et al., 2020).

In the present study, hysteresis appeared to quantitatively depend on the *cultivar:CI* (or color band) combination. For example, the correlation between R mean pixel values and oil in Koroneiki and Maiatica showed no hysteresis compared to other cultivars (**Figure 4**), anticipating it could be a good predictor. In contrast, the R color band was found to be the best predictor only in Maiatica (*R* ^2^ = 0.93, **Supplementary Figure S6**), while in Koroneiki other *CIs* (i.e., NG, GLI) had an overall better prediction capability (**Figure 13**, **Supplementary Figure S4**). Considering the differential changes of the seasonal R, G, and B mean pixel values, the application of additive linear models in the form of *Y ~ R+G+B* would be substantial in phenotyping oil concentration even in the case of hysteresis. This was the case in all analyzed cultivars (**Table 1**) showing that the model variance was able to explain up to 96% of the total variance, similarly to the prediction accuracy achieved using ANN (Montanaro et al., 2023). However, in a model having more than one predictor, the stability of coefficients might be corrupted if predictors are more related to each other than to the response variable; hence the use of a reduced version of the model is advisable (Chatterjee and Simonoff, 2013). In line with this, analysis of the variance inflation revealed the collinearity existing between predictors (i.e., VIF>5) in Fasola, Frantoio, Koroneiki and Leccino cultivars (**Table 1**). Hence, a reduced version of the model (*Y ~ R+B*, or *Y ~ G+B*) was proposed for these cultivars in **Table 2** to counteract the collinearity issue even if reducing the accuracy at least in some cultivars. That is, comparing these cultivars suffering collinearity in **Tables 1** and **2**, it can be highlighted that the simplified model increased the AIC by approx. 10-20% and reduced the *R* ^2^ by approx. 6-12%. Hence, minimising the number of predictors would at the same time minimise the risk of collinearity. Moreover, it is implicit in the VIF formula that models employing only one predictor (e.g., *Y ~ CI*, Equation 2) have no risk of collinearity. In line with this, the recombination of the R, G, and B generating the various *CIs* to be used as predictors would at the same time keep the advantage of multiple image information (i.e., color bands) avoiding the potential instability of model coefficients due to collinearity. In line with this, several *CIs* that were almost entirely derived from a previous study (Montanaro et al., 2023) were examined. The justification of the formulation of these *CIs* is grounded on existing correlative information between changes in R, G, and B and the occurrence of changes in pigments concentration, fatty substances accumulation, sugar concentrations, etc. (see Montanaro et al., 2023).

**Table 1:** Mean ( ± SE) of parameters determined for the predictors of the model *Y~ Red + Green + Blue* after five iterations performed over five random subsets of the training dataset in each olive cultivars.

	Arbequina	Fasola	Frantoio	Koroneiki	Leccino	Maiatica
coeff_(Intercept)	2.71 ± 0.16	-0.76 ± 0.77	8.54 ± 0.24	8.91 ± 0.66	28 ± 0.42	-11.76 ± 1.01
coeff_Red	0.28 ± 0	0.06 ± 0.04	0.37 ± 0.02	0.46 ± 0	0.33 ± 0.01	0.52 ± 0
coeff_Green	-0.25 ± 0	-0.1 ± 0.01	-0.32 ± 0.01	-0.44 ± 0.01	-0.28 ± 0	-0.18 ± 0.01
coeff_Blue	0.15 ± 0.01	0.46 ± 0.03	0.16 ± 0.01	0.2 ± 0.01	-0.26 ± 0.01	-0.12 ± 0
pval_(Intercept)	0.27 ± 0.03	0.62 ± 0.14	0.04 ± 0.01	0.29 ± 0.03	0 ± 0	0.11 ± 0.03
pval_Red	0 ± 0	0.46 ± 0.16	0 ± 0	0 ± 0	0 ± 0	0 ± 0
pval_Green	0 ± 0	0 ± 0	0 ± 0	0 ± 0	0 ± 0	0 ± 0
pval_Blue	0 ± 0	0 ± 0	0.03 ± 0.01	0.03 ± 0.01	0 ± 0	0.01 ± 0
AIC	113.35 ± 1.92	116.61 ± 2.28	153.37 ± 0.59	137.45 ± 0.55	122.97 ± 0.91	79.57 ± 0.98
VIF_Red	3.4 ± 0.08	**22.7 ± 0.68**	**7.29 ± 0.23**	2.76 ± 0.05	**22.89 ± 0.62**	2.19 ± 0.04
VIF_Green	2.13 ± 0.05	**14.45 ± 0.23**	**5.93 ± 0.18**	**8.23 ± 0.33**	**19.09 ± 0.47**	1.26 ± 0.02
VIF_Blue	1.93 ± 0.05	**7.07 ± 0.55**	2.29 ± 0.06	**6.07 ± 0.2**	2.19 ± 0.07	2.1 ± 0.03
*R* ^2^	0.92 ± 0.02	0.91 ± 0.02	0.83 ± 0.01	0.85 ± 0.01	0.88 ± 0.01	0.96 ± 0.01
MAE_response	109.33 ± 0.58	154.83 ± 0.88	292.35 ± 2.05	128.47 ± 0.94	128.45 ± 0.47	207.64 ± 1.26
RMSE.glm_response	119.05 ± 0.59	169.28 ± 1.21	316.1 ± 1.84	140.97 ± 1.21	141.48 ± 0.53	226.48 ± 1.82

Characters in bold indicate VIF>5.

**Table 2:** Mean ( ± SE) of parameters determined for the predictors of the reduced model *Y~ Green + Blue* (Fasola, Frantoio, Leccino) and *Y~ Red + Blue* (Koroneiki) after five iterations performed over five random subsets of the training dataset in various olive cultivars.

	Fasola	Frantoio	Koroneiki	Leccino
coeff_(Intercept)	0.45 ± 0.3	10.53 ± 0.5	-25.28 ± 0.1	25.61 ± 0.48
Coeff_Red	NA	NA	0.360 ± 0.0	NA
coeff_Green	-0.08 ± 0	-0.17 ± 0	NA	-0.1 ± 0
coeff_Blue	0.51 ± 0	0.38 ± 0.01	± 0	-0.08 ± 0.01
pval_(Intercept)	0.72 ± 0.1	0.04 ± 0.01	0 ± 0	0 ± 0
pval_Red	NA	NA	-0.110	NA
pval_Green	0 ± 0	0 ± 0	0 ± 0	0 ± 0
pval_Blue	0 ± 0	0 ± 0	0.1 ± 0.01	0.31 ± 0.05
AIC	116.12 ± 2.66	167.18 ± 0.99	153.16 ± 0.36	146.43 ± 0.33
VIF_Red	NA	NA	2.03 ± 0.03	NA
VIF_Green	1.02 ± 0	1 ± 0	NA	1.63 ± 0.03
VIF_Blue	1.02 ± 0	1 ± 0	2.03 ± 0.03	1.63 ± 0.03
*R* ^2^	0.93 ± 0.02	0.78 ± 0.03	0.74 ± 0.01	0.81 ± 0.01
MAE_response	154.72 ± 0.91	292.58 ± 1.99	128.53 ± 0.93	128.59 ± 0.45
RMSE.glm_response	169.13 ± 1.24	316.49 ± 1.85	141.28 ± 1.19	141.84 ± 0.59

NA, Not Applicable.

The number of possible RGB-derived indexes is relatively large making their selection a critical step also when used as input features of ANN requiring additional computational efforts. For example, in Montanaro et al. (2023), the *CIs* were selected after a PCA- or SPCA-based pre-processing with the purpose to minimise the risk of overfitting embedded in ANNs (Warne et al., 2004).

In the present study, new selection criteria were introduced relying on the quantification of the hysteresis generated by the correlation between oil concentration and *CIs*. Hence, a value to the hysteretic oil concentration in response to each *CI* was attributed using the *Hyst* index (Equation 4). In parallel with this, the coefficient of correlation was determined over the same distribution. The **Figure 7** reports the paired *Hyst* and ρ values determined over all the cultivars and *CIs* revealing that the highest coefficient of correlation (i.e., ρ close to -1 or 1) corresponded to the highest hysteresis (1 or -1). Such correspondence was consistent across the whole dataset as showed by the **Figure 8**. In this study, it was reported the hysteretic response of oil concentration across six cultivars and up to 35 *CIs* integrating current knowledge in image-based fruit phenotyping in olive. The variability of *Hyst* values recorded across cultivars and *CIs* is likely to be attributable to different ripening time of these cultivars and their interaction with the environment, but this remain to be specifically examined.

To simultaneously account for the impact of *CI* on both hysteresis and ρ, the *FI* has been proposed to support the selection of *CI* (**Figure 9**). Based on the *FI*, specific models and *CI* were identified allowing a prediction accuracy (*R* ^2^) comparable to that achieved in a previous paper adopting ANN (Montanaro et al., 2023).

In the present work, the GPI was employed to simultaneously account within the same cultivar for multiple accuracy indicators (*R* ^2^, MAE, RMSE) as reported by Despotovic et al. (2015). Analysis revealed that within each cultivar there is a good agreement between the GPI-based rank of models/predictors and that of the *FI*. That is, the predictors with the highest *FI* (see red dots in **Figure 13**) rank 1st-2nd according to GPI. Cosidering that “The more the accuracy of the model the higher the value of the GPI” (Despotovic et al., 2015) results support the suitability of *FI* in selecting the best predictors. However, in Arbequina and Koroneiki cultivar a criticism might rise because the highest *FI* did not match the top GPI-based rank of predictors. To explain this apparent discrepancy, it could be considered that 7/8 (Koroneiki) and 6/7 *FIs* (Arbequina) falling in the top quintile had a relatively short range of *FI* (i.e., 1.86:1.93) (**Figure 9**).

This paper reports for Mediterranean traditional and SHD olive systems the seasonal pattern of oil accumulation contributing to expand existing information on this topic. This study also examined the application of a GLM to predict the oil concentration using R, G, and B and RGB-based colorimetric indexes. For this purpose, this paper provides a new pipeline to achieve the selection of the most suitable predictor. The pipeline includes the quantification of the hysteretic correlation between the response variable and the *CIs* adopting for the first time in olive crop science a procedure similar to that used in other research fields (e.g., electronics, human health). The predictions have an overall accuracy comparable to that of ANN models requiring additional computational efforts.

## Data Availability

The raw data supporting the conclusions of this article will be made available by the authors, without undue reservation.
