# Supplementary material for: Predicting oil accumulation by fruit image processing and linear models in traditional and super high-density olive cultivars
Source: Front Plant Sci. 2024 Oct 25;15:1456800. doi: 10.3389/fpls.2024.1456800 (PMC11589486; doi:10.3389/fpls.2024.1456800)
Supplement: Supplementary file 1 [file DataSheet1.pdf]

Supplementary Material

Predicting oil accumulation by fruit image processing and linear models in traditional and super high-density olive cultivars

Giuseppe Montanaro[*], Antonio Carlomagno, Angelo Petrozza, Francesco Cellini, Ioanna Manolikaki, Georgios Koubouris, Vitale Nuzzo

*** Correspondence:** Giuseppe Montanaro: giuseppe.montanaro@unibas.it

1 Supplementary Figures and Tables

1.1 Supplementary Figures

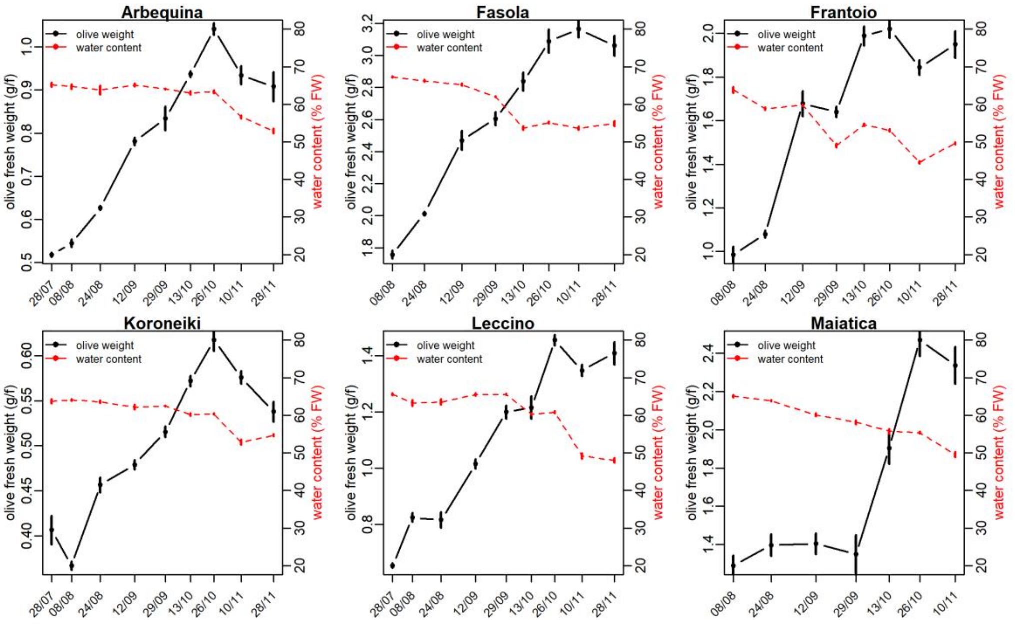

Supplementary Figure 1. Seasonal trend of mean fresh weight of intact single fruit (g/f), and of water content (%FW) recorded in various olive cultivars. Vertical bars represent ±SE and are visible when larger than symbol.

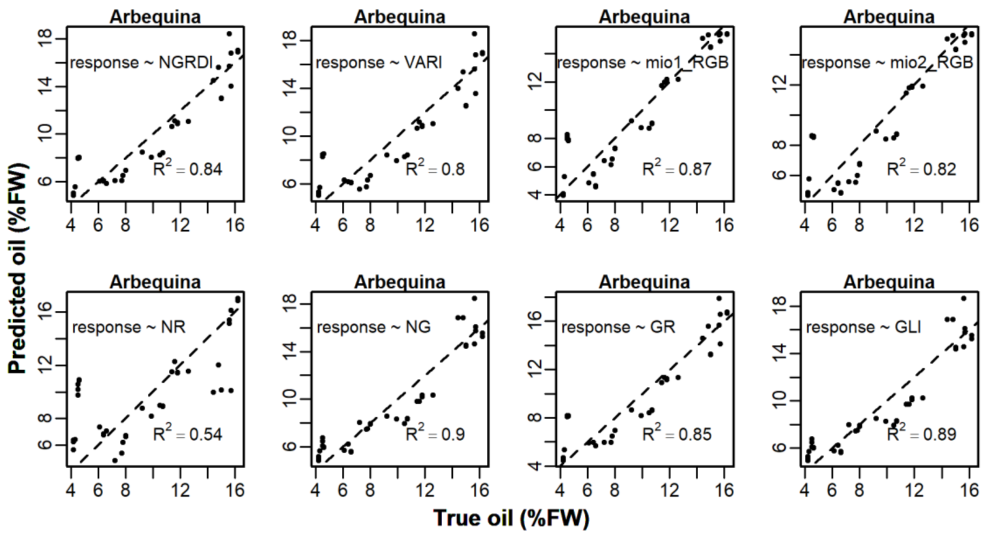

Supplementary Figure 2. Scatterplot of the *predicted* ~ *true* oil (%FW) correlation achieved in Arbequina after the five iterations of the model *response* ~ *CI* (colorimetric index) selected base on fitness index. The specific CI in reported in each panel. Values of the R^2 is the mean determined over the five iterations. The dashed line represents the 1:1 straight line.

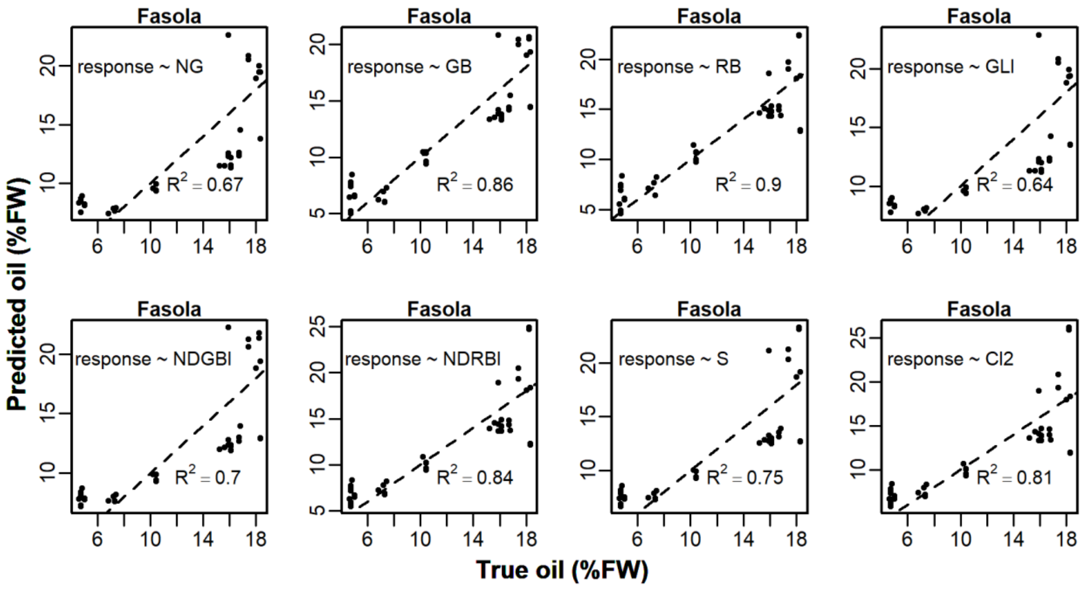

Supplementary Figure 3. Scatterplot of the *predicted* ~ *true* oil (%FW) correlation achieved in Fasola after the five iterations of the model *response* ~ *CI* (colorimetric index) selected base on fitness index. The specific CI in reported in each panel. Values of the R^2 is the mean determined over the five iterations. The dashed line represents the 1:1 straight line.

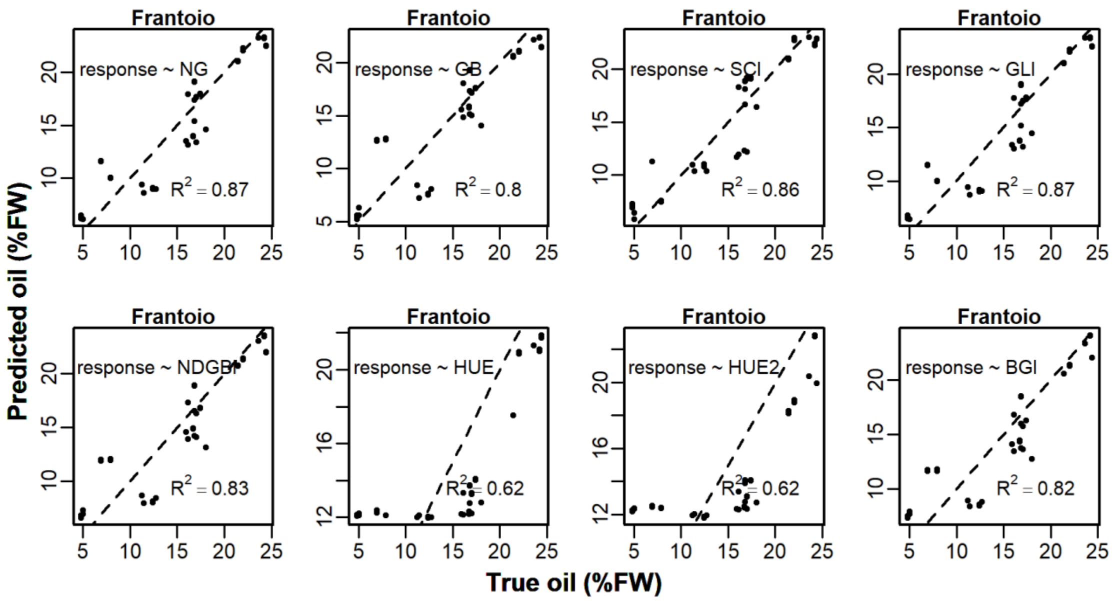

Supplementary Figure 4. Scatterplot of the *predicted ~ true* oil (%FW) correlation achieved in Frantoio after the five iterations of the model *response ~ CI* (colorimetric index) selected based on fitness index. The specific CI in reported in each panel. Values of the R^2 is the mean determined over the five iterations. The dashed line represents the 1:1 straight line.

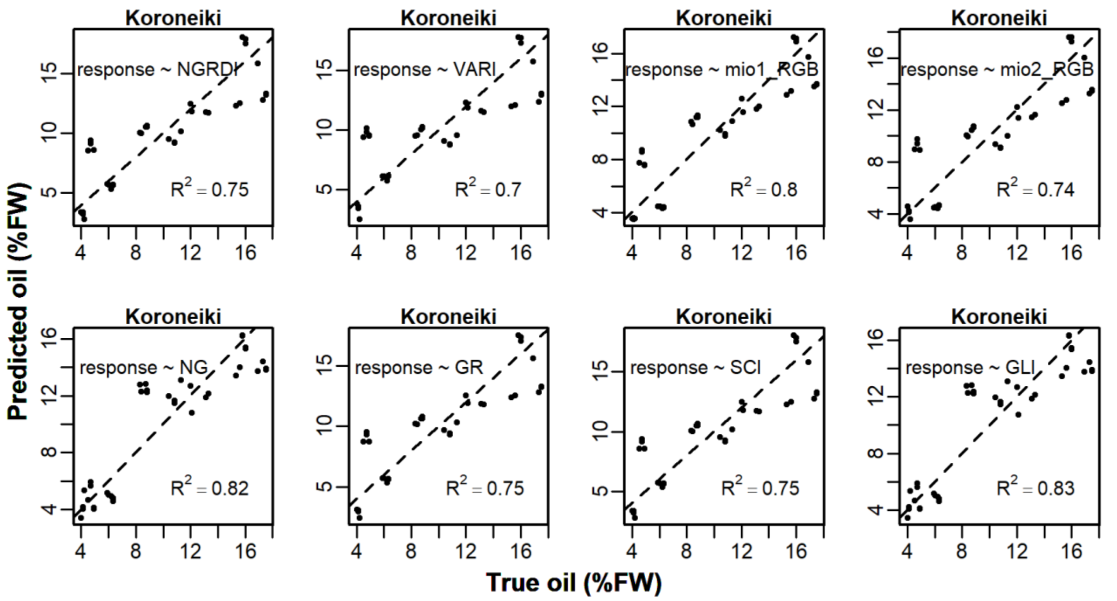

Supplementary Figure 5. Scatterplot of the *predicted ~ true* oil (%FW) correlation achieved in Koroneiki after the five iterations of the model *response ~ CI* (colorimetric index) selected base on fitness index. The specific CI in reported in each panel. Values of the R^2 is the mean determined over the five iterations. The dashed line represents the 1:1 straight line.

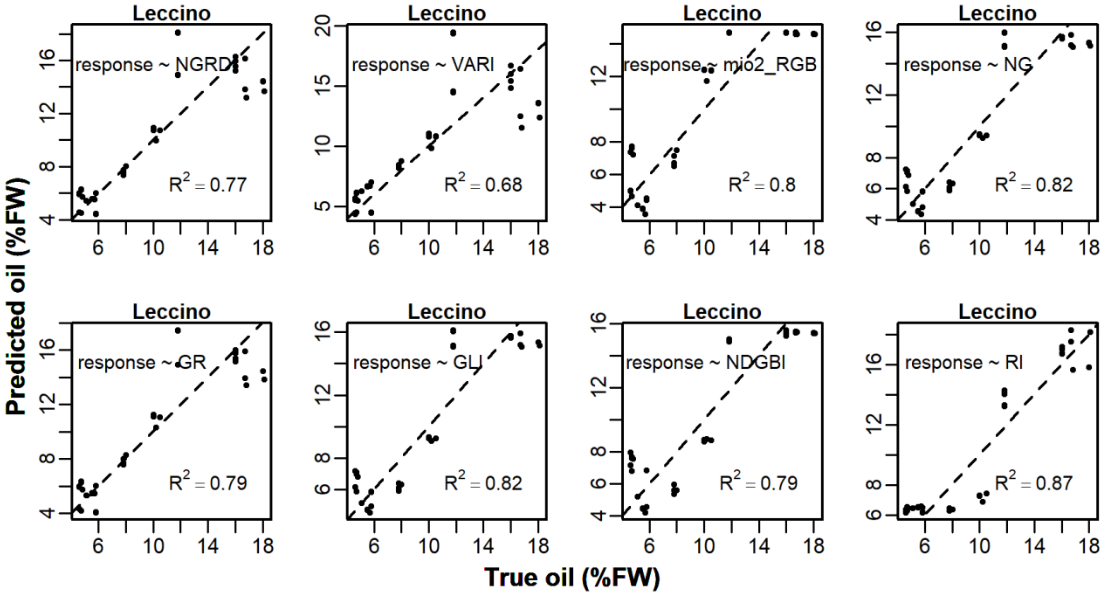

Supplementary Figure 6. Scatterplot of the *predicted* ~ *true* oil (%FW) correlation achieved in Leccino after the five iterations of the model *response ~ CI* (colorimetric index) selected base on fitness index. The specific CI in reported in each panel. Values of the R^2 is the mean determined over the five iterations. The dashed line represents the 1:1 straight line.

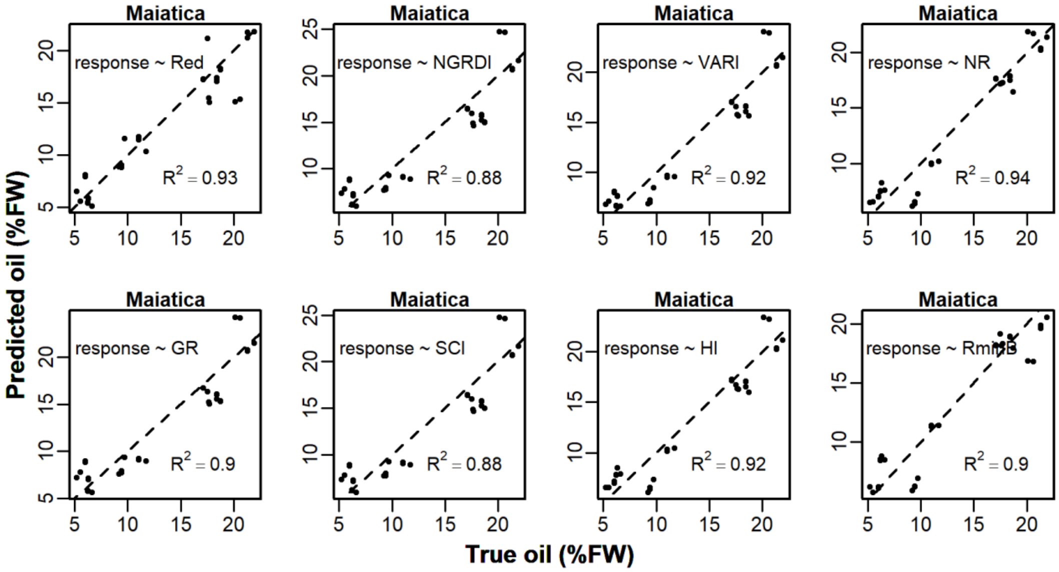

Supplementary Figure 7. Scatterplot of the *predicted* ~ *true* oil (%FW) correlation achieved in Maiatica after the five iterations of the model *response ~ CI* (colorimetric index) selected base on fitness index. The specific CI in reported in each panel. Values of the R^2 is the mean determined over the five iterations. The dashed line represents the 1:1 straight line.

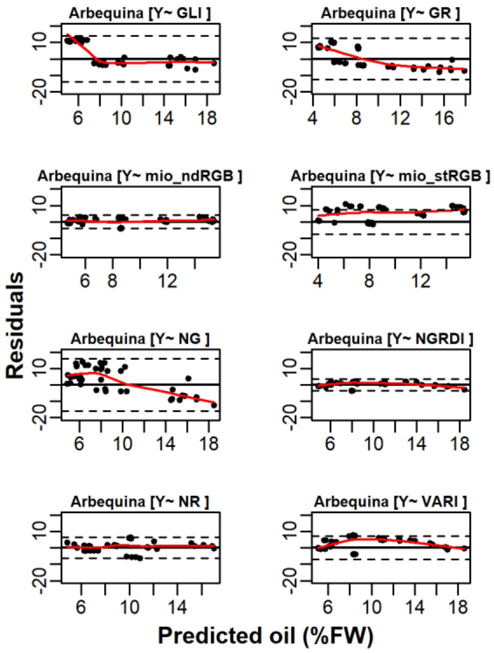

Supplementary Figure 8. Residuals plots of the Y ~ CI models for the estimates of oil concentration in Arbequina cultivar. The bold horizontal lines represent zero in the ordinates values, dashed horizontal lines define the interval of the ±2 standard deviations calculated on the residuals, and the red one is the locally-weighted scatterplot smoothing line.

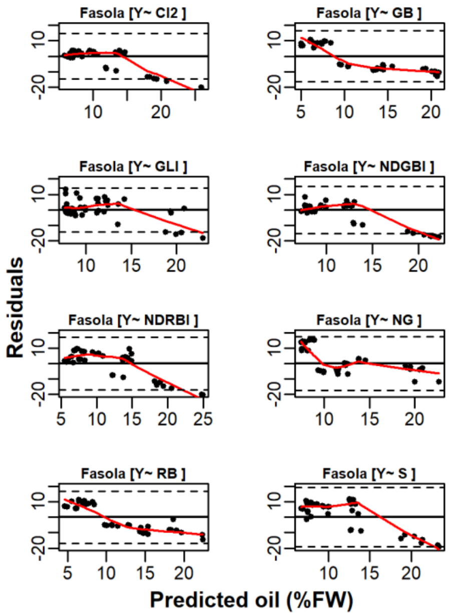

Supplementary Figure 9. Residuals plots of the Y ~ CI models for the estimates of oil concentration in Fasola cultivar. The bold horizontal lines represent zero in the ordinates values, dashed horizontal lines define the interval of the ±2 standard deviations calculated on the residuals, and the red one is the locally-weighted scatterplot smoothing line.

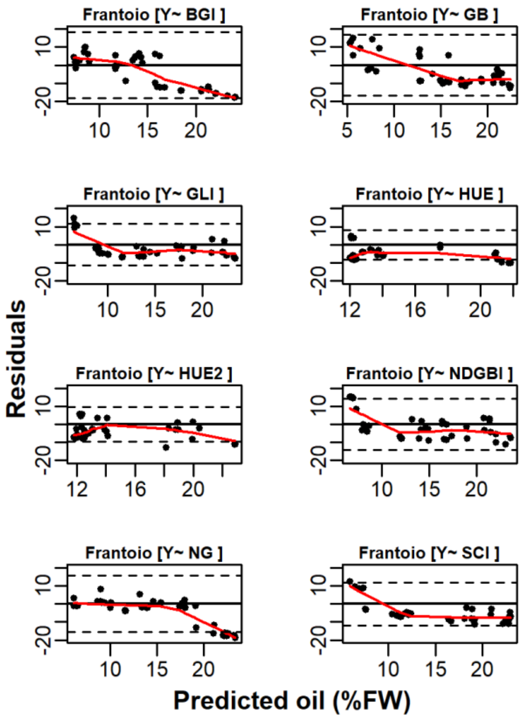

Supplementary Figure 10. Residuals plots of the Y ~ CI models for the estimates of oil concentration in Frantoio cultivar. The bold horizontal lines represent zero in the ordinates values, dashed horizontal lines define the interval of the ±2 standard deviations calculated on the residuals, and the red one is the locally-weighted scatterplot smoothing line.

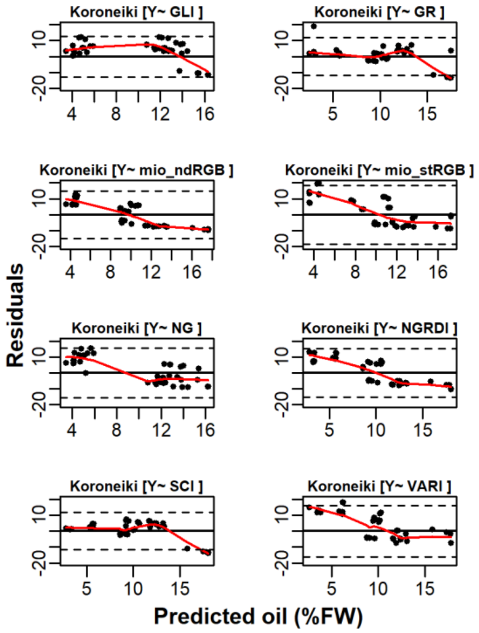

Supplementary Figure 11. Residuals plots of the Y ~ CI models for the estimates of oil concentration in Kotoneiki cultivar. The bold horizontal lines represent zero in the ordinates values, dashed horizontal lines define the interval of the ±2 standard deviations calculated on the residuals, and the red one is the locally-weighted scatterplot smoothing line.

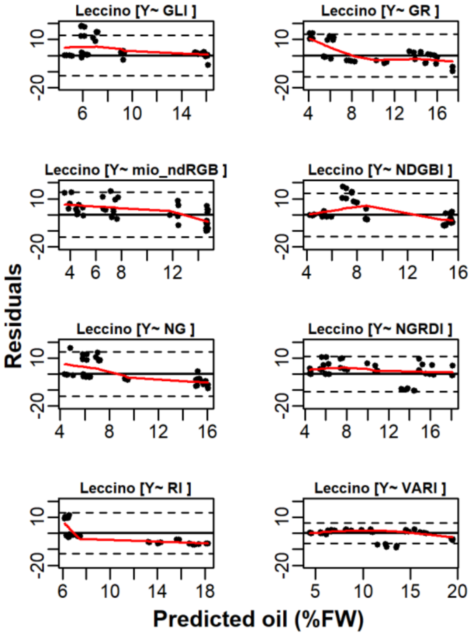

Supplementary Figure 12. Residuals plots of the Y ~ CI models for the estimates of oil concentration in Leccino cultivar. The bold horizontal lines represent zero in the ordinates values, dashed horizontal lines define the interval of the ±2 standard deviations calculated on the residuals, and the red one is the locally-weighted scatterplot smoothing line.

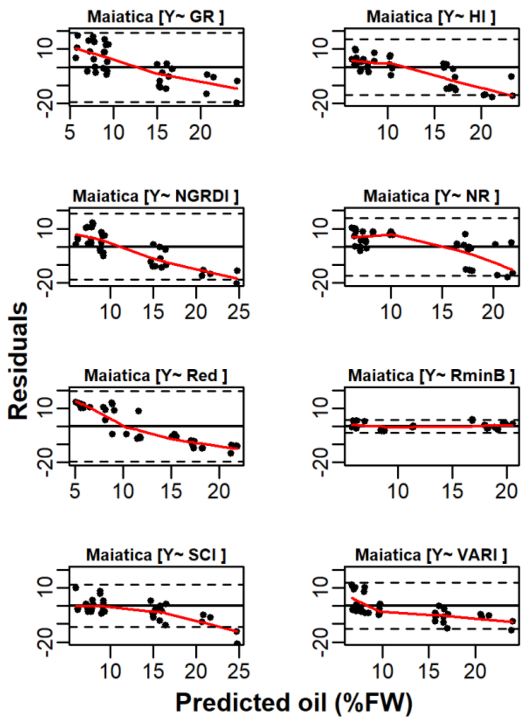

Supplementary Figure 13. Residuals plots of the Y ~ CI models for the estimates of oil concentration in Maiatica cultivar. The bold horizontal lines represent zero in the ordinates values, dashed horizontal lines define the interval of the ±2 standard deviations calculated on the residuals, and the red one is the locally-weighted scatterplot smoothing line.

1.2 Supplementary Tables

Table 1. Colorimetric indexes determined from mean pixel value of R, G, and B for details see Montanaro et al., (2023).

Index	Formula	Index	Formula	Index	Formula
R	Red	GLI	(2*G-R-B)/(2*G+R+B)	GRAY	0.299*R+0.587*G+0.114*B
G	Green	HI	(2*R-G-B)/(G-B)	CI2	(R-B)/R
B	Blue	NGRDI	(G-R)/(G+R)	mio1_RGB	(R-G)^2/B
NR	R/(R+G+B)	NDGBI	(G-B)/(G+B)	mio2_RGB	(R-G)^2/sqrt(B)
NG	G/(R+G+B)	NDRBI	(R-B)/(R+B)	RI	R^2/(B*G^3)
NB	B/(R+G+B)	I	R+G+B	RminB	R-B
GB	G/B	S	((R+G+B)-3*B)/(R+G+B)	RplusB	R+B
RB	R/B	VARI	(G-R)/(G+R-B)	RplusG	R+G
GR	G/R	HUE	atan(2*(B-G-R)/30.5*(G-R))	RminG	R-G
BI	sqrt((R^2+G^2+B^2)/3)	HUE2	atan(2*(R-G-R)/30.5*(G-B))	GminB	G-B
BIM	sqrt((R*2+G*2+B*2)/3)	BGI	B/G	BplusG	B+G
SCI	(R-G)/(R+G)	L	R+G+B/3		
